# Using Landscape Genetics Simulations for Planting Blister Rust Resistant Whitebark Pine in the US Northern Rocky Mountains

**DOI:** 10.3389/fgene.2017.00009

**Published:** 2017-02-10

**Authors:** Erin L. Landguth, Zachary A. Holden, Mary F. Mahalovich, Samuel A. Cushman

**Affiliations:** ^1^Division of Biological Sciences, University of MontanaMissoula, MT, USA; ^2^U.S. Department of Agriculture Forest ServiceMissoula, MT, USA; ^3^U.S. Department of Agriculture Forest Service, Northern, Rocky Mountain, Southwestern and Intermountain RegionsMoscow, ID, USA; ^4^U.S. Department of Agriculture Forest Service, Rocky Mountain Research StationFlagstaff, AZ, USA

**Keywords:** assisted migration, CDMetaPOP, computer simulations, ecological niche modeling, genotype-environment associations, landscape genomics, wind resistance

## Abstract

Recent population declines to the high elevation western North America foundation species whitebark pine, have been driven by the synergistic effects of the invasive blister rust pathogen, mountain pine beetle (MPB), fire exclusion, and climate change. This has led to consideration for listing whitebark pine (WBP) as a threatened or endangered species under the Endangered Species Act, which has intensified interest in developing management strategies for maintaining and restoring the species. An important, but poorly studied, aspect of WBP restoration is the spatial variation in adaptive genetic variation and the potential of blister rust resistant strains to maintain viable populations in the future. Here, we present a simulation modeling framework to improve understanding of the long-term genetic consequences of the blister rust pathogen, the evolution of rust resistance, and scenarios of planting rust resistant genotypes of whitebark pine. We combine climate niche modeling and eco-evolutionary landscape genetics modeling to evaluate the effects of different scenarios of planting rust-resistant genotypes and impacts of wind field direction on patterns of gene flow. Planting scenarios showed different levels for local extirpation of WBP and increased population-wide blister rust resistance, suggesting that the spatial arrangement and choice of planting locations can greatly affect survival rates of whitebark pine. This study presents a preliminary, but potentially important, framework for facilitating the conservation of whitebark pine.

## Introduction

Whitebark pine (WBP; *Pinus albicaulis*) is one of the most intensively studied North American conifers, in part due to its unique relationship with the grizzly bear (*Ursus arctos horribilis*), Clark's nutcracker (*Nucifraga columbiana*), and over 20 other wildlife species (Lorenz et al., 2008), which depend on its seeds for food; thus it is considered a keystone and foundation species in high elevation forests within its range. Thus, recent declines associated with the spread of mountain pine beetle (MPB; *Dendroctonus ponderosae*), and the introduced invasive fungal pathogen white pine blister rust (WPBR; *Cronartium ribicola*) have led to consideration for listing the species as threatened under the Endagered Species Act in 2010 (Federal Register 2010), intensifying interest in developing strategies for its conservation and management (see recent reviews by Keane et al., 2012, 2016).

One of the primary threats associated with WBP decline is WPBR-an invasive fungal pathogen introduced to the Pacific Northwest of North America around 1910 (Brar et al., 2015). WPBR affects the productivity and distribution of WBP by forming cankers, which girdle branches and boles, resulting in reduced cone production and increased tree mortality. It has since spread to five-needle pine species across the United States.

Genetic blister rust resistance was first identified in small samples of open-pollinated families by Bingham (1972) and Hoff et al. (1980). A larger trial of 110-seed sources later established the efficacy of identifying, propagating, and deploying blister rust resistant seedlings (Mahalovich et al., 2006). While major gene resistance has not been found in WBP, three resistance mechanisms exhibit as single-gene recessives. The no-spot and needle shed resistance mechanisms are present in very low frequencies (<1%), while the short shoot resistance mechanism is present in low frequency (5.2 percent, Mahalovich *in prep*). In the US Northern Rockies, offspring of over 1300 phenotypic selections are under evaluation in support of active restoration by planting proven, rust-resistant seedlings which have a combination of no-spot, needle-shed, bark reaction and shoot resistance mechanisms (Mahalovich and Dickerson, 2004; Greater Yellowstone Coordinating Committee whitebark pine Subcommittee, 2011; Keane et al., 2012, 2016).

Advances in landscape genetics and population genomics provide a robust means to predict the effects of landscape structure and climatic gradients on genetic structure, population connectivity, and adaptive genetic variation (Manel and Holderegger, 2013; e.g., Shryock et al., 2015). Recently developed simulation modeling tools provide effective means to link landscape patterns to gene flow and adaptive evolutionary processes to predict genetic characteristics of the population across its range under current and potential future conditions (Scribner et al., 2016). Simulation models offers several important benefits for landscape genomic research (Landguth et al., 2015). For example, simulation modeling can be used to predict how a system or its behavior will change if certain processes or parameters are altered. This is particularly relevant for predicting the effects of environmental change on a system, or for evaluating the likely outcomes of various management scenarios.

Our primary objective for this study was to develop a simulation modeling framework for assessing the connectivity of WBP across the US Northern Rocky Mountains and to assess the potential adaptive significance of genetic blister rust resistance. Specifically, we first developed climate niche models for WBP and WPBR distributions. Then, we used these models with an eco-evolutionary landscape genetics model to simulate demographic and genetic (i.e., demogenetic; Frank et al., 2011) responses with and without the presence of white pine blister rust. We conducted simulations that introduced a resistant gene for WPBR and simulated potential planting strategies with this genotype. We also tested the influence of wind field directionality on the ability of pollen to disperse rust-resistant genes through the landscape. Finally, future WBP landscape genetics studies are discussed, including planting strategies with WPBR resistant individuals in conjunction with adaptive simulation modeling experiments.

## Materials and methods

### Whitebark pine regeneration and white pine blister rust suitability model

We developed correlative niche models (CNM; aka species distribution or habitat suitability models; Thuiller et al., 2005; Elith and Leathwick, 2009) for WBP and WPBR using occurrence records (presence and absence) to develop a probabilistic model of occurrence based on statistical relationships with climatic, topographic and biophysical variables. One criticism of CNM's applied to long-lived tree species is that they typically correlate adult occurrence records with climate data from relatively short time periods (i.e., 30–50 years). This means that at some locations, an adult tree >300 years old may have established under a very different climate than the one being used to represent its climatic suitability. Recent studies have suggested using juvenile rather than adult occurrences to provide a more realistic characterization of the relationship between a species and a suitable climatic period (Lenoir et al., 2009; Zhu et al., 2011; Bell et al., 2014; Dobrowski et al., 2015). In this study, we used juvenile (<130 mm diameter) occurrence records from Forest Inventory and Analysis (FIA) plot data on all public lands occurring within US Forest Service Northern Region. As predictors we developed a suite of high resolution (240 m) temperature, climatic water balance, and snow distribution models. Gridded data were extracted using the raster library in the R software environment using bilinear interpolation of the four nearest neighbor cells at each FIA plot location. Additional details on the development of the climatic water balance data are provided in Appendix 1. Details about the CNM for WBP and WPBR occurrence are provided in Appendixs 2, 3, respectively.

### Whitebark pine simulation model

We used CDMetaPOP (Landguth et al., 2016) to simulate how the presence of WPBR and individuals with resistance to WPBR influence WBP demogenetics. CDMetaPOP is a landscape-level, spatially-explicit, and individual-based eco-genetic model of meta-population processes. CDMetaPOP simulates demogenetic processes as interactions between individuals located across a number of “patches” (hereafter, stands) containing meta-populations. Individuals within a stand are assumed to share a common environment (e.g., carrying capacity, temperature). Within each stand, a class (age/stage/size) structure is used to simulate complex stochastic demographic processes, while movement of individuals (i.e., seeds and pollen) between stands is controlled as a function of spatially-explicit landscape resistance or permeability surfaces (e.g., directional wind resistance to movement). More simply stated, a landscape is populated with stands, which in turn are populated with individual trees. At the stand level, individuals undergo growth, reproduction, migration, and mortality, and the resulting genetic processes are simulated over time at the individual-tree level. For more detailed information on the processes simulated in CDMetaPOP, see the user manual (https://github.com/ComputationalEcologyLab/CDMetaPOP).

Our WBP model required parameterization of a number of species-specific processes (see Appendix 4, Figure A4.1 and Table A4.1). After initialization of the model (e.g., stands, stage structure, and genetics), pollen dispersal (age 0) occurs during the summer. Then, cones from the current year's pollination/fertilization event emerged on each tree and seeds dispersed in the fall (age 1). Over winter, stage-structured density dependent mortality was implemented as a function of each stand's carrying capacity (K). Growth of all individuals and establishment of new mature individuals (age 20+) occurred by spring and the additional WPBR mortality on mature individuals was implemented at this time. More detailed methods with data sources used to parameterize the model are outlined below and in Appendix 4, Table A4.1.

#### Stands, carrying capacity, age, and size classes

**Figure 1:**
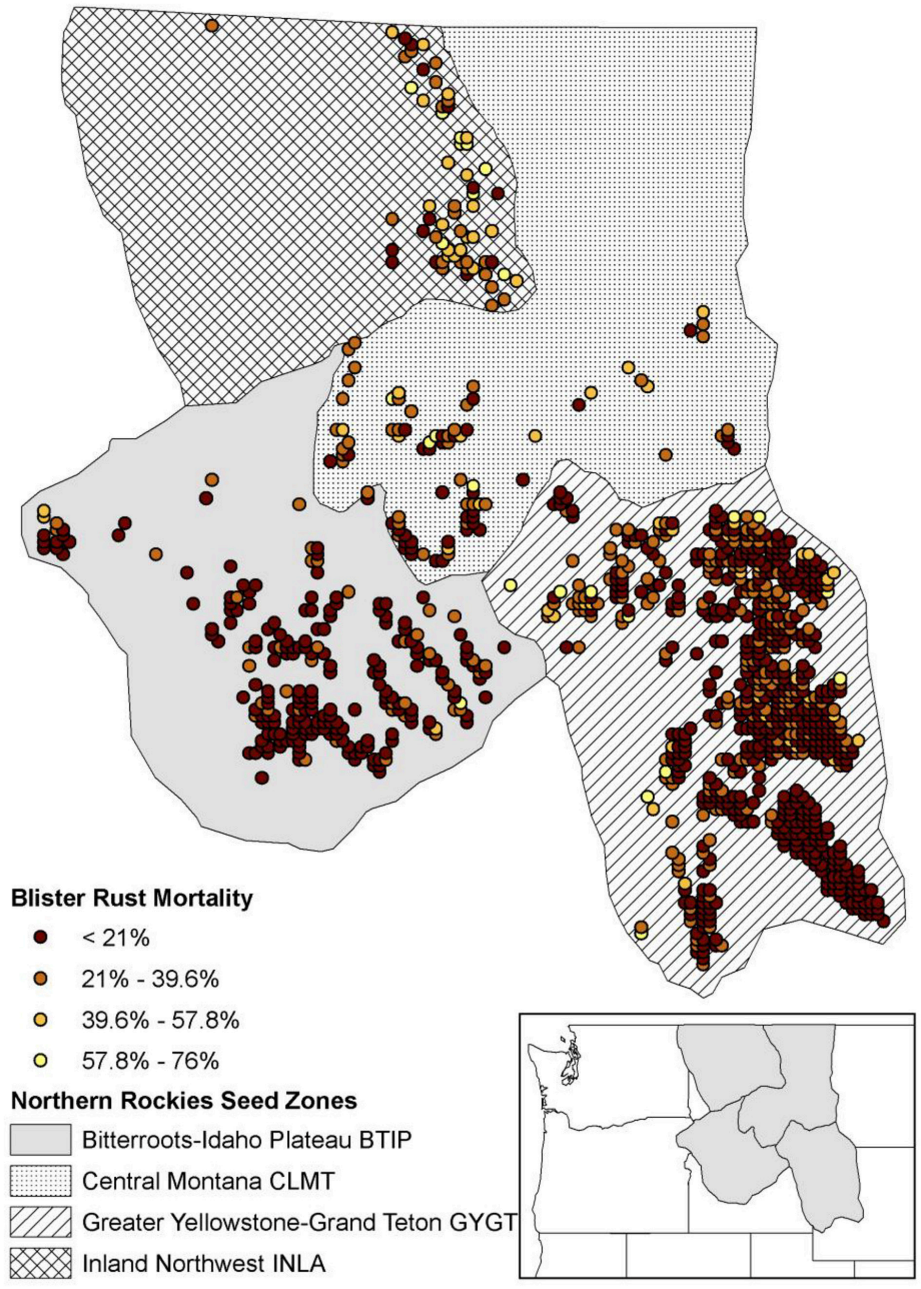
**Study area defined by the northern Rockies seed zones (Mahalovich and Hipkins, 2011) with initial 1059 simulated stand locations**. WPBR relative spatial selection mortality shown for each stand.

The WBP simulations were constrained to an extent in the US Northern Rockies that was delineated a priori by four zones (i.e., “seed zones”; Mahalovich and Hipkins, 2011; Figure 1). The extent contained 1059 initial spatially-delineated stand locations separated by at least 5 km. These WBP stands were designated by selecting all cells with >0.5 probability of WBP suitability, as predicted by the CNM described above (see Section Whitebark Pine Regeneration and White Pine Blister Rust Suitability Model and Appendix 2). For simplicity, we assumed a carrying capacity of 100 trees at each stand location.

We initialized the model at time = 0 with a random distribution of 500 age classes (Burns and Barbara, 1990). We ran the model without genetic exchange for an initial 25 years to allow the age distribution to stabilize, and then began genetic exchange (see next section). We defined age 0 “individuals” as fertilization events, which 12 months later emerged as age 1 cones producing seeds for dispersal. An annual increment of 0.2 cm diameter at breast height (DBH) (Keane et al., 2007) was used to grow each individual tree. As trees progressed through each size class, size-linked parameters (e.g., probability of mortality, probability of maturation, and fecundity) varied (Appendix 4).

#### Neutral and adaptive genetics

We initialized each individual's neutral genotypes with allele frequency files that match the frequency observed in each seed zone (Mahalovich and Hipkins, 2011), comprised of 16 loci with at most nine polymorphic alleles per locus. We did not consider mutation, which is reasonable considering the short simulation time period. In addition, we added a bi-allelic adaptive locus and assumed that only one gene confers resistance to WPBR (e.g., Kinloch et al., 1999; Lui et al., 2016). We initialized this selection-driven locus at time = 25 years with 0.01 and 0.99 frequency for the first and second allele, respectively. Any individual homozygous at the first allele (i.e., AA) in this selection-driven locus was assumed to have a selective advantage against blister rust infection.

This simple single-locus selection model was chosen because major gene resistance between the host species and pathogen has not been found in WBP (Bingham, 1983; Kinloch and Dupper, 2002), and much of our understanding of blister rust gene resistance comes from interior western white pine (*Pinus monticola*; Kinloch et al., 1999) and recently, Rocky Mountain white pine (*Pinus flexilis*; Lui et al., 2016). Thus, we assumed the blister rust resistance mechanisms acting in WBP are comparable to these species. Furthermore, the interior western white pine (*Pinus monticola*) blister rust screening program (Bingham, 1983; Mahalovich, 2010) serves as the basis for WBP blister rust screening trials (Bingham, 1983; McDonald and Hoff, 2001; Mahalovich et al., 2006). While there are other presumed single-gene recessive traits present in low frequency in blister rust screening trials (Mahalovich et al., 2006), the blister rust resistance trait chosen for modeling was the short shoot fungicidal reaction (Hoff and McDonald, 1971) due to the higher frequency of these genotypes in blister rust screening trials from 1999 to 2015 (Mahalovich, unpublished data). This resistance mechanism involves necrosis at the base of an infected needle fascicle bundle; thus, normal canker growth is halted and the branch and tree stem remains disease-free.

#### White pine blister rust resistance and mortality

CDMetaPOP implements natural selection analogously to the adaptive-, or fitness-landscape of allele frequencies as originally envisioned by Wright (1932). This functionality enables extension of landscape genetic analyses to explicitly investigate the links between gene flow and selection in complex landscapes at the level of the individual (see Landguth et al., 2012a). We used WPBR occurrence (see Section Whitebark Pine Regeneration and White Pine Blister Rust Suitability Model) values at each stand as a proxy for differential mortality applied to mature trees only (e.g., WPBR occurrence of 0.5 would produce a 50% mortality at that stand; Figure 1). WPBR mortality rates in each stand were implemented based on the genotype of each individual and increased survival was associated with individuals that had *AA* in the selection-driven locus, which varied depending on the simulation scenario (see section Simulation Scenarios and Analysis). This allowed us to model evolution of WPBR resistance based on a single locus under selection with a single genotype being selected for.

#### Maturation and fecundity

Mature individuals were defined as those of age 20 and greater. Although WBP may typically take longer to reach maturity when growing on poorer sites or at higher elevations (e.g., Krugman and Jenkinson, 1974; Mahalovich unpublished data), we used a lower bound of 20 years to allow for more generations in the model (Fire Effects Information System; http://www.fs.fed.us/database/feis/plants/tree/pinalb/all.html accessed September, 2015). We implemented a size-based fecundity model to determine the number of seeds produced at a given basal area per stand following the individual tree DBH conversion to basal area: Basal Area = 0.00007854 ^*^ DBH^2^. To obtain a size-based seed production per individual tree, we used the value of 500 cones per 1 basal area (m^2^/ha; Barringer et al., 2012) multiplied by 20 seeds per cone. Although cone and seed production varies spatially and temporally in our study area (Owens et al., 2008), no masting was considered and we assumed lower bound estimates (e.g., as low as 10 seeds per cone; Pigott, 2012) to reduce computational time.

#### Mortality

In order to isolate the effects of WPBR mortality, we only considered density-independent mortality based on class-based mortality probabilities. We applied a 99% probability of mortality to age 0 class to mimic 1% seed survival (DeMastus, 2013). We implemented a cumulative 35% probability of survival for age classes 1–15 (Izlar, 2002). Trees age 500 and older were assigned 25% probability of survival, which allowed for occasional long-lived trees (i.e., >500 years) given the length of the simulation time. If a stand reached K, then a random removal of excess individuals was conducted (e.g., Balloux, 2001).

#### Reproduction, pollen dispersal, and wind directionality

Reproduction within and across stands was monecious with selfing allowed. We considered two hypotheses for pollen movement in the summer months. Our first hypothesis assumed pollen moved according to a null model of isolation-by-distance: probability of pollen dispersal to a respective female cone locations was a function of the inverse-square Euclidean distance (Landguth and Cushman, 2010) with a 50% maximum study area distance threshold (450 km). Because pollen dispersal is governed by wind patterns, we also considered a second hypothesis that included directional movement with respect to prevailing wind direction (i.e., isolation-by-distance and wind). Thirty-year average (1979–2010) mean annual average wind direction was calculated from the North American Regional Reanalysis (NARR; Mesinger et al., 2006). Using the landscape connectivity program, UNICOR (Landguth et al., 2012b), we created asymmetrical costs for traversing with and against wind direction for all pairwise stand-to-stand locations. UNICOR creates a graph of a given resistance surface, which allows start and end node locations to find shortest paths on the resistance surface (i.e., Dykstra's algorithm). Given a wind direction map (and ignoring vector magnitude), a resultant vector was created in the 8-Moore neighborhood to weight direction in the graph creation. This produced an added cost resulting from the resultant vector calculation and when a path was traversing from a point and against wind direction, producing an asymmetrical cost distance matrix.

#### Cone/seed dispersal

Age 1 cones from the previous year were dispersed from individual trees (e.g., Clark's nutcracker, a bird which disperses and caches WBP seeds) following an isolation-by-distance movement pattern similar to pollen dispersal: probability of cone dispersal to a new stand location was a function of the inverse-square Euclidean distance with a 30 km maximum distance threshold (Lorenz et al., 2011). This produced the majority of cones staying in the same stand or nearest neighbor stands (i.e., dropping near parent tree) with occasional longer distance cone dispersal (e.g., Clark's Nutcracker). In addition to 1% seed survival (DeMastus, 2013), the ability for a seed to establish in a new stand location was determined based on resource availability (i.e., carrying capacity not exceeded in the destination stand).

### Simulation scenarios and analysis

**Figure 2:**
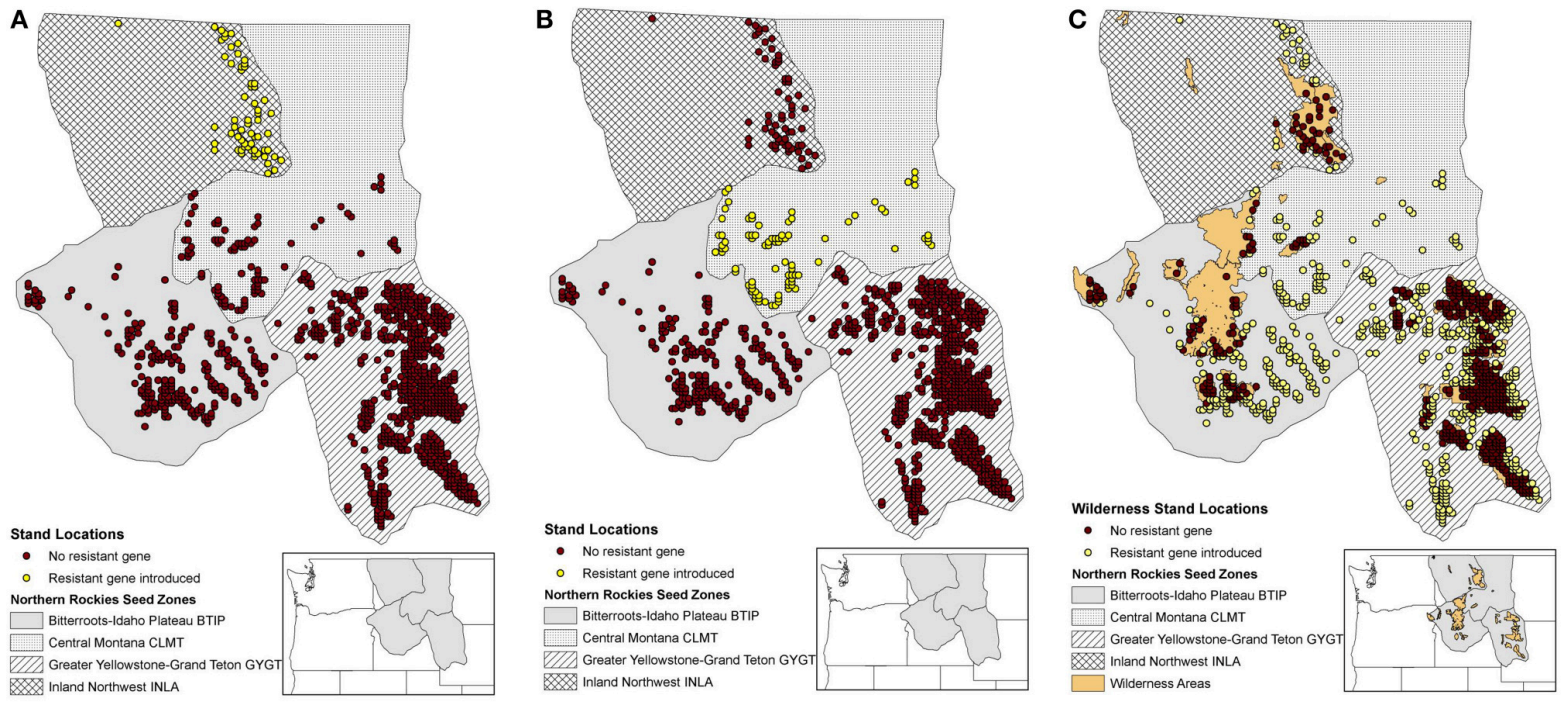
**Study area considered using the northern Rockies seed zones (Mahalovich and Hipkins, 2011) with initial 1059 stand locations. (A)** WPBR resistant gene introduced in INLA zone stands (yellow dots). **(B)** WPBR resistant gene introduced in CLMT zone stands (yellow dots). **(C)** WPBR resistant gene introduced in stands (yellow dots) outside of wilderness areas (brown dots).

We conducted two blocks of simulation scenarios. The first block of simulations was used to help understand the added influence of WPBR mortality with and without an introduced gene that was resistant to WPBR. The second block of simulations was used to look at different spatial patterns for planting individuals with a resistance to WPBR. The spatially planting strategies we explored included planting in two regions (seed zones), as well as a broader distribution of planting across the entire extent outside of wilderness areas (Figures 2A–C). Each block compared pollen dispersal simulations for isolation-by-distance and directional pollen dispersal via wind. Table 1 lists each block and respective scenario.

**Table 1:** **Simulation scenarios (WPBR–white pine blister rust)**.

**Block Name**	**Scenario Name**	**Description**
Block 1: WPBR mortality and resistance	No mortality	The null model in which no WPBR mortality considered
	All mortality	All stand locations applied the added WPBR mortality (Figure 1) regardless of genetic makeup.
	Resistant gene in all zones	All stand locations applied the added WPBR mortality (Figure 1). One genotype assumed to confer resistance to WPBR.
Block 2: WPBR resistance by planting strategy	Resistant gene in INLA zone	All stand locations applied the added WPBR mortality. One genotype assumed to confer resistance to WPBR only in the most northern zone (INLA; Figure 2A).
	Resistant gene in CLMT zone	All stand locations applied the added WPBR mortality. One genotype assumed to confer resistance to WPBR only in a central zone (CLMT; Figure 2B).
	Resistant gene in non-wilderness	All stand locations applied the added WPBR morality. One genotype assumed to confer resistance to WPBR only outside of wilderness areas (Figure 2C).

We ran simulations for 130 years, with the first 25 years considered “burn-in” for the population dynamics and age distributions to stabilize. We plotted mean population abundance, allelic diversity, and heterozygosity for all stands and for each block scenario. We used 10 replicate simulation runs to assess variation in each metric. For a spatial representation of genetic differentiation, we calculated an overall pairwise genetic differentiation (*G*_ST_) across all loci using the method of Nei (1973) and for each pair of zones at specified year *t* = 100.

## Results

### Whitebark pine and white pine blister rust maps

**Figure 3:**
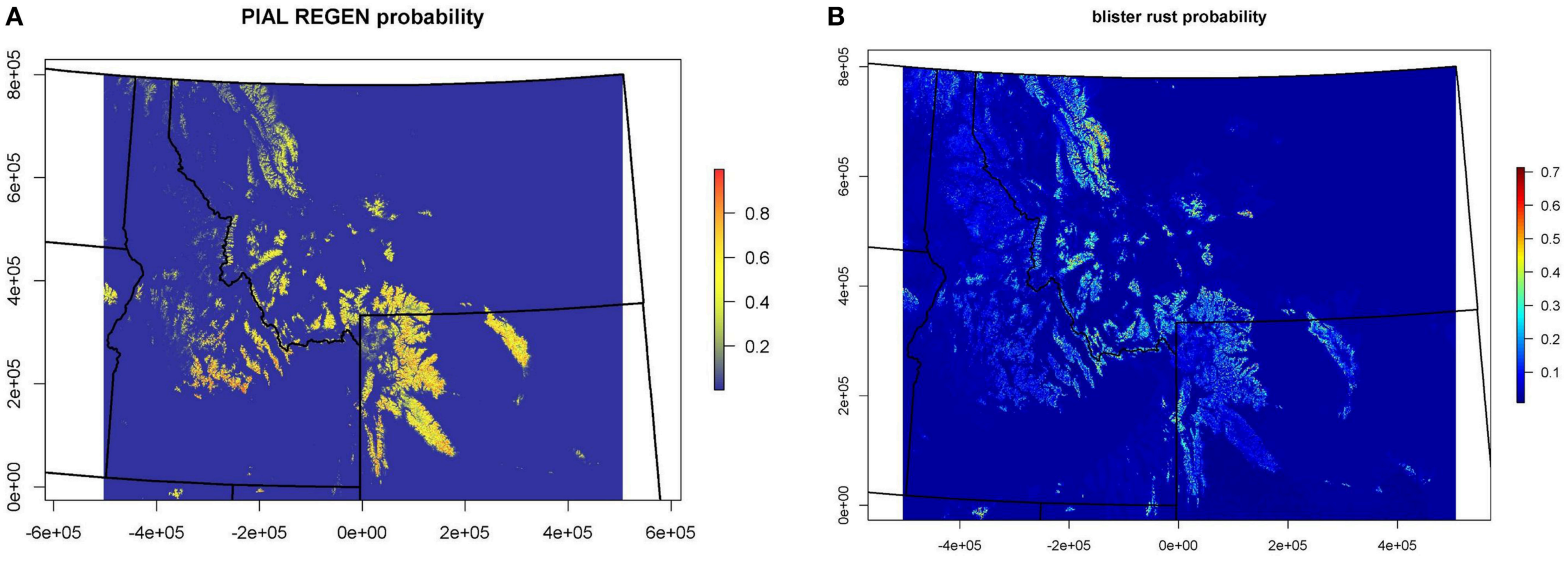
**Probability of occurrence maps for (A)** whitebark pine and **(B)** white pine blister rust.

Results from the CNM for the presence or absence of juvenile WBP and WPBR within US Forest Service Northern Region are shown in Figure 3. See Appendix 2, 3 for supporting documentation on models. The distribution of juvenile WBP was reasonably well predicted by biophysical predictors, and presence or absences of juveniles was correctly classified at 92% of the forest inventory plots (Table A2.1). Mean maximum daytime temperature, followed by mean annual water balance deficit (unit of measure), were the strongest predictors in the WBP model. The model predicts that WBP occurs with highest probability at high elevation, cold sites with moderate to low water balance deficit. The distribution of WPBR was moderately well explained by climatic and biophysical predictors, with an overall classification accuracy of 81%.

### White bark pine landscape demogenetic simulations

**Figure 4:**
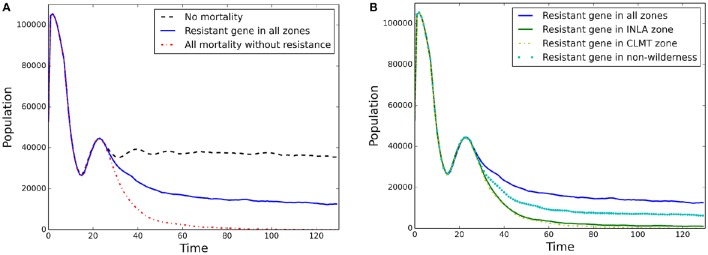
**Population abundance through time for each scenario in (A)** Block 1 and **(B)** Block 2.

Overall population mean abundances (i.e., all stands) for each block of scenarios are shown in Figure 4 for the simplest model of isolation-by-distance with no wind resistance included for pollen dispersal. Block 1 “No mortality” (Figure 4A black dashed line) shows stable population dynamics, while in the “All mortality” scenario the population declined smoothly to close to 0 by time 100 (Figure 4A red dash-dotted line). The introduction of a WPBR resistant gene for all individuals at every stand while still applying WPBR differential mortality led to stable population sizes of approximately 1/4th of the “No mortality” scenario (Figure 4A blue solid line).

Block 2 scenarios are shown in Figure 4B. Planting of individuals with resistance in the two different zones resulted in near extirpation of WBP (central CLMT zone; Figure 4B yellow dash-dotted line, and northern INLA zone; Figure 4B green line). Figure 4B also shows the scenario for the more widely distributed planting outside of Wilderness areas (Figure 4B cyan dotted line), which produced a stable population abundance at approximately 1/2 of the “Resistant gene in all zones” (Figure 4B blue solid line). Similar results for mean stand growth rate are shown in Appendix 4 (Figures A4.2a,b).

**Figure 5:**
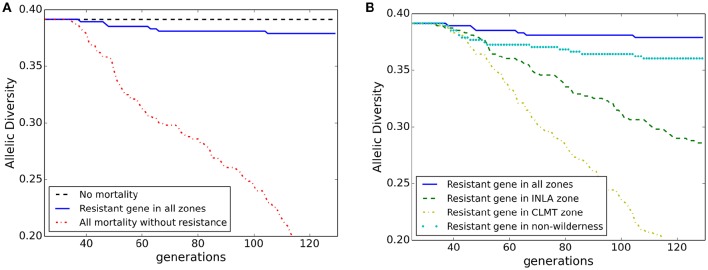
**Allelic diversity through time for each scenario in (A)** Block 1 and **(B)** Block 2.

Overall population mean allelic diversity is shown in Figure 5 for the model of isolation-by-distance with no wind resistance included for pollen dispersal. The decline in allelic diversity revealed patterns similar to those of the population abundance graphs. The allelic diversity in the null model of no spatial differential mortality remained relatively constant at 0.39 (Figure 5A black dashed line). In the extreme scenario where WPBR was applied to every stand, allelic diversity steeply declined to 0.2 (Figure 5A red dashed-dotted line) and in the scenario in which WPBR resistant genotypes were planted in every stand, allelic diversity remained close to the null model (0.38; Figure 5A blue solid line). However, there was a greater loss in allelic diversity with the central (CLMT) zone planting scenario (0.2; yellow dash-dotted line; Figure 5B) compared to the northern (INLA) zone planting scenario (0.3; green dashed line), despite equivalent population abundance, showing how genetic diversity may be more sensitive to spatial planting than overall abundance. Furthermore, planting of resistant WPBR individuals in a continuous distribution across the analysis extent produced higher allelic diversity numbers than the zone-specific planting (Figure 5B cyan dotted line). Similar results are shown for heterozygosity in Appendix 4 (Figures A4.3a,b).

**Figure 6:**
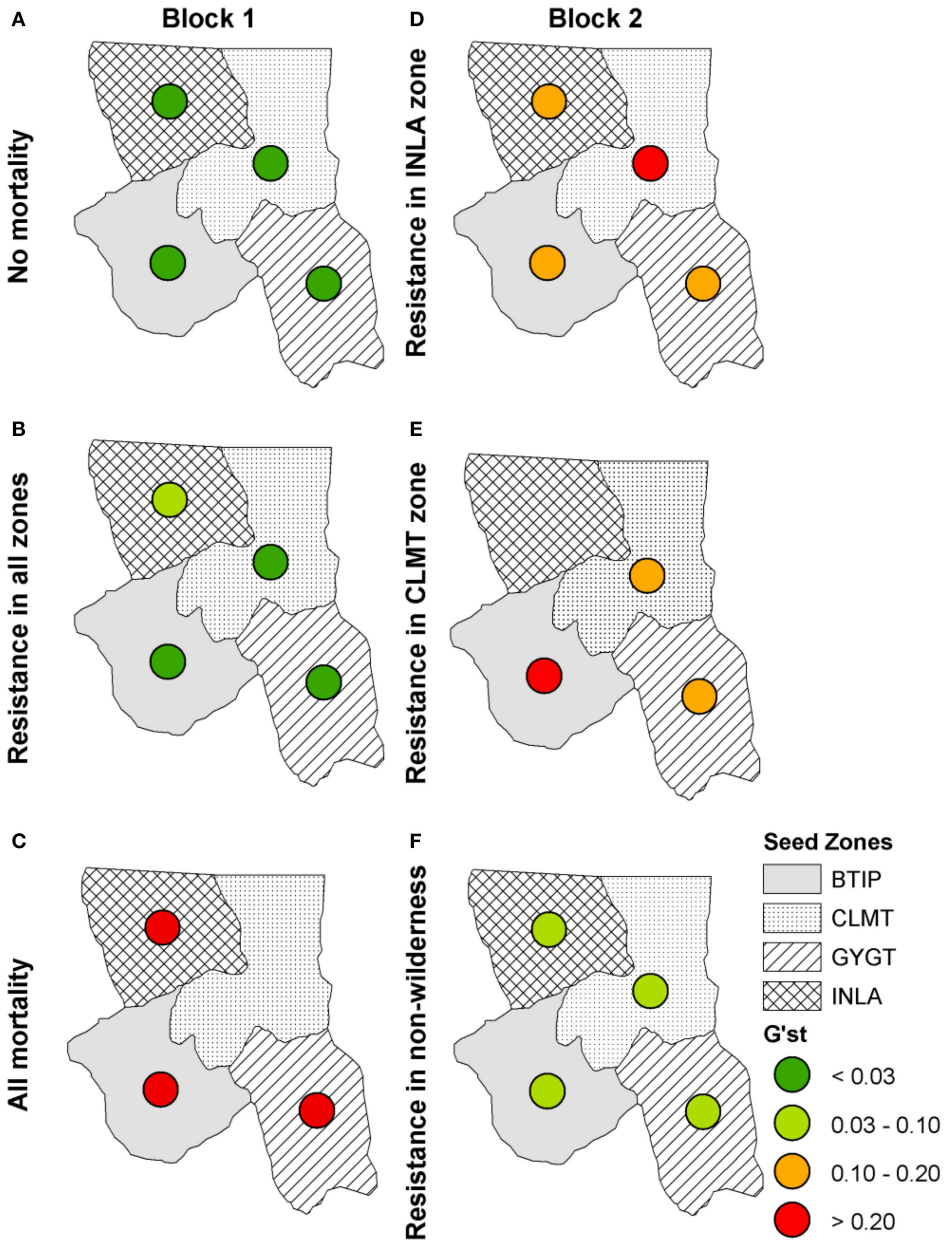
**Isolation-by-distance: *G'*_ST_ values for each seed zone at year 100 for Block 1 scenarios: (A)** the null scenario “No mortality,” **(B)** the scenario in which a resistant gene was introduced, **(C)** the scenario in which all stands receive WPBR mortality (“All mortality”) and for Block 2 scenarios: **(D)** the scenario in which a resistant gene was introduced in only the INLA zone, **(E)** the scenario in which a resistant gene was introduced in the CLMT zone only, **(F)** the scenario in which a resistant gene was introduced outside of wilderness areas only.

Genetic differentiation for each zone is shown in Figure 6 for time 100 for the model of isolation-by-distance with no wind resistance. The “No mortality” scenario (Figure 6A) shows little difference in genetic differentiation through time. However, as WPBR mortality is applied, genetic differentiation increases, with the largest differentiation in the “All mortality” scenario (Figure 6C). In fact, with the “All mortality” scenario, the CLMT zone becomes extirpated. The uniform introduction of a resistant WPBR gene produced patterns of genetic differentiation among zones similar to the “No mortality” scenario, with the exception of the INLA zone showing slightly higher differentiation (Figure 6D). The right panel in Figures 6C–F shows the Block 2 scenarios that varied spatial planting strategies for resistant genes to WPBR. Genetic differentiation increased under all planting strategies, with local extirpation occurring with the CLMT zone-specific scenario (Figure 6E). Genetic differentiation for non-Wilderness area planting of resistant genes only slightly increased (Figure 6F) from the null model of “No mortality” (Figure 6A).

**Figure 7:**
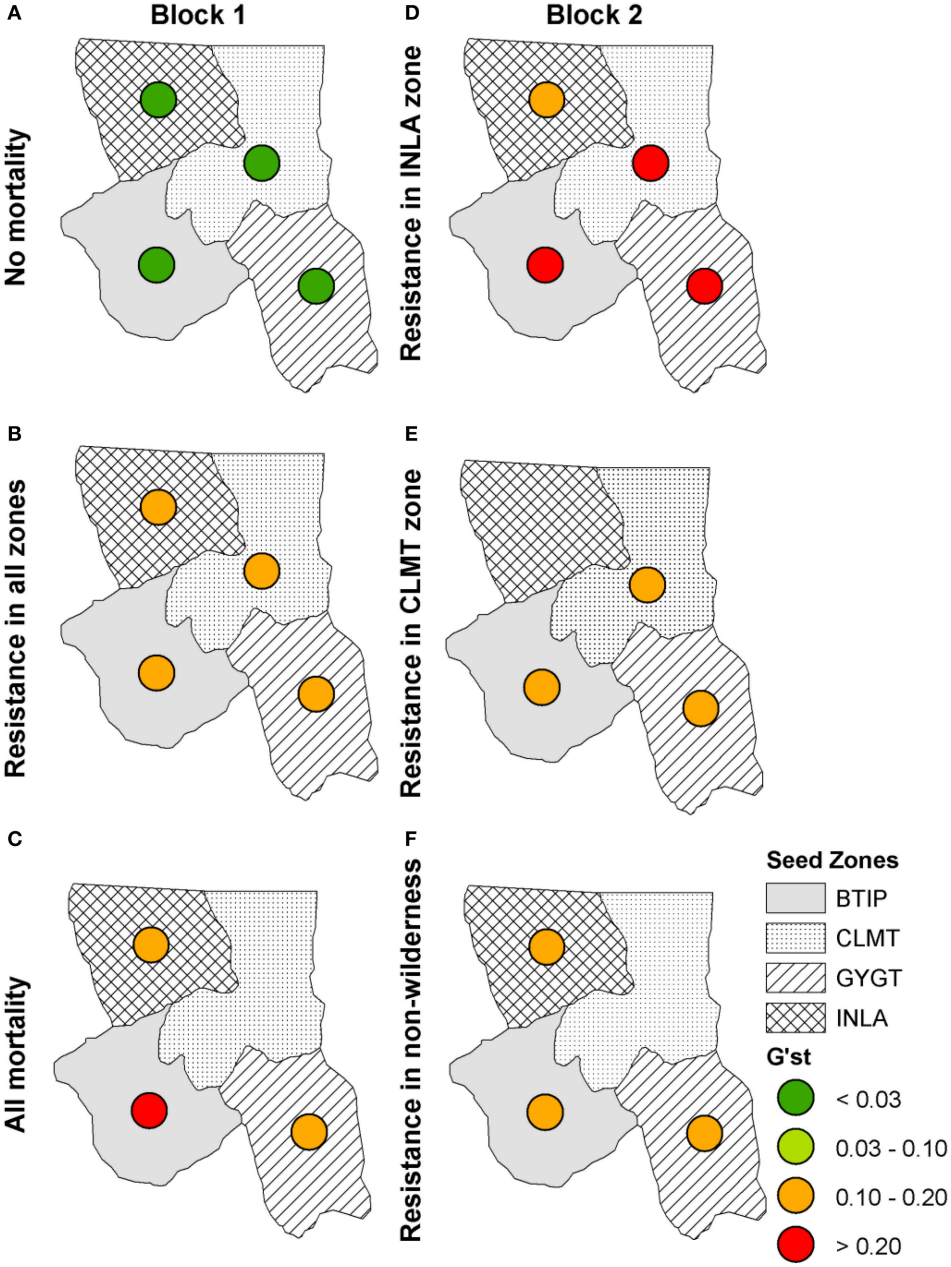
**Isolation-by-distance with directional wind resistance: *G'*_ST_ values for each seed zone at year 100 for Block 1 scenarios (left panel): (A)** “No mortality,” **(B)** “blister rust resistance in all zones,” **(C)** “All mortality” and Block 2 scenarios (right panel): **(D)** “Resistance in INLA zone,” **(E)** “Resistance in CLMT zone,” **(F)** “Resistance in non-wilderness.”

When we included the effects of directional wind resistance on pollen dispersal we see an overall increase in genetic differentiation across all scenarios (Figure 7) with the exception of the “No mortality” scenario (Figure 7A), which remained at the same level of genetic differentiation as with the model of just isolation-by-distance. We also see more local extirpation, in particular in the scenario in which individuals with WPBR resistance are only planted in non-Wilderness areas (Figure 7F). These simulations show that incorporating more realistic effects of spatial processes, such as wind resistance, reduces pollen dispersal capability, thus reducing the ability of resistance genes to propagate through the landscape.

## Discussion

The goal of this paper is to provide an example of integrating species distribution modeling with landscape genetic simulation of neutral gene flow and adaptive evolution. Our specific focus was on exploring the effects of different levels of pathogen lethality and gene flow on the evolution of blister rust resistance in WBP and the effectiveness of several scenarios of planting rust resistant genotypes of WBP in different spatial configurations. This is the first simulation experiment to examine local and regional demogenetic patterns to the placement of resistant individuals, and the first to quantify differences in adaptive evolutionary processes as a function of directional and isotropic resistance to dispersal.

We first presented climate niche models for WBP and WPBR distributions. We used the climate niche models with a new eco-evolutionary landscape genetics model to simulate demogenetic responses with and without the presence of the disease agent, white pine blister rust. These models allowed us to produce baseline null models of a ”healthy” disease-free system (e.g., Figure 4A black dashed line) with stable demographics and genetics and an extreme case of complete disease-ridden system (e.g., Figure 4A red dash-dotted line) with crashing demographics and genetics.

We then introduced individuals with a genotype that conferred resistance to WPBR and simulated potential planting strategies with this genotype in example zone-specific locations and across more broadly distributed areas across the study extent (i.e., outside of wilderness areas). This allowed us to quantify how much the introduction of disease resistant genotypes might mitigate the effects of WPBR and to evaluate this model systems sensitivity to the extent and pattern of introduction of disease resistant genotypes. Our results demonstrate that different patterns of planting resistant genotypes can influence genetic outcomes, and that genetic diversity and differentiation are more sensitive than population dynamics (Figure 5B compared to Figure 4B). Furthermore, planting of resistant WPBR individuals in a systematic distribution across the study area extent produced much higher allelic diversity numbers than more localized “clusters” (Figure 5B). A growing body of research has suggested that the loss of genetic diversity with increased disease may be a crucial mechanism driving population extinction risk (Whiteman et al., 2006). Thus, this finding could have additional important implications for management planning and suggests that strategies should focus on implementing broad-scale, spatially continuous introductions rather than focusing on concentrating planting of disease resistant genotypes in particular nodal populations (e.g., Oyler-McCance et al., 2013). However, this also implies potential logistical limitations to effective management of WBPR through introduction of disease resistant genes across broad regions. Specifically, for rust resistance to spread in a local population it must be introduced with sufficiently high frequency to not be rapidly lost through drift before it can spread through selection. This is more easily achieved through concentrated introductions in patches or zones. However, our results show that broad-scale, continuous introductions are needed to effectively mitigate population and genetic effects of WPBR. It is not clear whether resources could be sufficiently invested to implement such widely distributed planting at sufficient density to produce a lasting effect on the population.

Our results also show that large differences in predicted genetic differentiation are produced when models use simple isolation-by-distance assumptions as compared to when they implement more realistic spatial processes, such as isolation by resistance. Specifically, scenarios that incorporated the influence of wind resistance on the ability of pollen to disperse resistant genes through the landscape produced much higher rates of local extirpation along with higher genetic differentiation (Figure 7). These simulations showed that incorporating more realistic landscapes that control for movement, such as wind resistance, reduces pollen dispersal capability, thus reducing the ability of resistance genes to propagate through the landscape. This has important implications for spatial genomic and evolutionary modeling, most of which has to date utilized simple models of isolation-by-distance controlling gene flow (but see Forester et al., 2015). Our results show that it is essential to move this work into an explicitly landscape genomic framework in which gene flow is realistically driven by spatial patterns of landscape features that influence dispersal (such as wind fields in this case).

To produce reliable inferences about implications of adaptive variation, researchers must unambiguously determine whether markers for key adaptive traits, such as blister rust resistance, are under selection and identify the factors in the environment that drive that selection (Joost et al., 2013; Rellstab et al., 2015). This, however, remains a challenging task (see Vitalis et al., 2001; Luikart et al., 2003; Angeloni et al., 2011). For example, outlier detection methods will often detect signals of selection in markers that are not themselves under selection, but instead just linked to a gene that is (e.g., Jones et al., 2014). Moreover, when numerous regions of the genome are under divergent selection, outlier analyses can miss many regions that clearly are under selection (Michel et al., 2010). Further complications arise for the ability to detect adaptive loci when landscape configuration, dispersal ability, and selection strength intertwine (Forester et al., 2015), as well as the effects of sampling through design, replication, and resolution of markers (e.g., number of SNPs) (e.g., De Mita et al., 2013; Lotterhos and Whitlock, 2015). Developing methods for reliably identifying markers under selection is a major ongoing theme in landscape genomics research. Common garden experiments with reciprocal transplant of genotypes is a robust way to assess environmental selection (e.g., Whitham et al., 2006; Cushman, 2014) and can be readily extended to evaluate the interactions between environmental selection and pathogen resistance. For the simulation framework identified here to be truly useful to understand the potential of genetically mediated blister rust resistance to mitigate impacts on WBP populations, it will be important to identify the genetic mechanisms controlling resistance and how they may be linked to selection on other factors, such as drought and cold tolerance.

There are several lines of addition future work which should be explored to extend the scope of what we have presented here on the spatial dynamics of adaptation to the blister rust pathogen and the potential effectiveness of different strategies of planting resistant genotypes. First, this paper used a simple one-locus model of genotype-environment association. While this is a model that is widely used in theoretical evolutionary ecology (Coyne and Orr, 2004) and genotype-environment association testing (e.g., Jones et al., 2014; Forester et al., 2015) and applies to some proposed blister rust mechanisms (Kinloch et al., 1999), the majority of micro-evolutionary processes are likely mediated through polygenetic selection in which many loci each contribute relatively small fitness effects. This paper serves as an initial analysis of a simple classical model of one locus selection which provides insight. However, future modeling work should explore how the blister rust distribution and planting of resistant genotypes interacts within the context of multiple loci/allele selection, pleiotropy, and epistasis.

In addition, while this paper is the first to combine empirical data, experimentation, and large-scale population-wide simulation modeling, WBP and WPBR are complex systems that are still imperfectly understood and simulation models are a simplified representation of reality. Future studies should invest in improving how WBP and WPBR biology are represented in simulations (e.g., more realistic growth models or disease spread dynamics) and assess sensitivity and uncertainty in these systems. For example, simulations could explore the effects of habitat quality and density-dependent processes (Pfluger and Balkenhol, 2014) on the interaction between rust resistance and white bark pine population dynamics. In addition, simulation experiments, such as presented here can describe the processes affecting population and identify the conditions under which they have important influences. However, models without data are not compelling. It is essential to confront these models with empirical data on the actual patterns of genetic differentiation in complex landscapes, and to confirm the fitness relationships underlying these patterns in experimental studies, such as common gardens (Cushman, 2014).

## Author contributions

EL, ZH, MM, and SC designed research. EL ran simulations. EL and ZH performed the analyses. EL, ZH, MM, and SC interpreted the data and wrote the manuscript.

### Conflict of interest statement

The authors declare that the research was conducted in the absence of any commercial or financial relationships that could be construed as a potential conflict of interest.
