# Supplementary material for: Using Landscape Genetics Simulations for Planting Blister Rust Resistant Whitebark Pine in the US Northern Rocky Mountains
Source: Front Genet. 2017 Feb 10;8:9. doi: 10.3389/fgene.2017.00009 (PMC5300977; doi:10.3389/fgene.2017.00009)
Supplement: Supplementary file 1 [file DataSheet1.DOCX]

# **Appendix 1. Development of high resolution topographically resolved climatic water balance grids for ecological modeling**

High spatial resolution (240 m) monthly climatic water balance (CWB) data were generated for the purpose of modeling the distribution of whitebark pine and blister rust occurrence. We used a Penman-Monteith based CWB model described by Dobrowksi et al. (2013) and a simple bucket model described by Lutz et al. (2010). Inputs required by the model include monthly mean temperature, relative humidity, wind speed, solar radiation, soil water holding capacity and precipitation. In regions of complex topography like the Northern Rockies, all of these inputs vary at relatively fine scales. Solar insolation varies as a function of aspect position, which drives fine-scale differences in surface air temperature. Wind speed, one of the strongest drivers of moisture demand varies as a function of aspect position and is generally lower, on average in sheltered valley bottoms. Soil depth is typically greater in valley bottoms, where smaller soil particles tend to accumulate over time, and is generally shallower on steep, rocky slopes. When we consider these variables together, they point to the potential for significant variation in moisture demand and availability over relatively small distances.

In order to better represent the fine-scale topoclimatic variation in energy demand and moisture retention across the region, all inputs to the current CWB model were downscaled using a combination of physics-based or statistical models. The inputs used and their sources are shown in Table A1.1. Monthly mean relative humidity and precipitation data were derived from PRISM 800 meter monthly data and resampled to 240 meters using cubic resampling. Monthly mean surface air temperature was calculated using daily datasets developed by Holden et al. (2015), Monthly mean solar insolation grids were derived from the daily mean insolation grids described by Holden et al. 2015).

Table A1.1. Input variables with their source and native spatial resolution used for modeling climatic water balance.

| **Variable** | **description** | **Source** | **Resolution** | **Units** |
| --- | --- | --- | --- | --- |
| Tavg | Monthly mean temperature | Holden et al. (2015) | 240 m | °C |
| Solar | Monthly mean solar insolation | Holden et al. (2015) | 240 m | W/m^2^ |
| Windspeed | Monthly mean wind speed | WindNinja (this study) | 240 m | m/s |
| AWS | Soil water storage capacity | gSSURGO / (this study) | 240 m | cm |
| RH | Monthly mean relative humidity | PRISM 800 m data | 800 m | % |
| PRCP | Monthly mean precipitation | PRISM 800 m data | 800 m | mm |

Monthly mean wind speed maps for the study domain were generated using the WindNinja model (Forthofer 2007). WindNinja is a physics-based wind speed model that can be parameterized with either point sources (e.g. RAWS stations) or weather model data. Here, we used data from the North American Regional Reanalysis dataset (NARR; Messinger et al. 2006) to parameterize mean daily windspeed runs from 1979-2012. Due to memory limitations, the model was run on 1x1 degree tiles, with a 15 km buffer around each tile edge. Daily runs were then aggregated to monthly grids. Then, comparisons with data from RAWS stations were used to correct a modest positive bias in the resulting wind speed outputs.

Gridded soil water holding data (AWC) were developed for the continental United States at 240 m resolution using the gridded SSURGO database and the soil data access query tool provided through the R library soilDB (Beaudette and Skovlin 2015). Large portions of United States have no available soils information in gSSURGO. Most missing data occurs in the western US, and several major wilderness areas in the Northern Rockies region have no available data. In order to develop spatially complete soils datasets, we used statistical models to develop relationships between available gridded soils data and topographic and vegetation indices. The fitted models were then used to predict water storage capacity at cells with missing data. We used the machine learning algorithm Random Forest (Breiman 2001) to develop models for AWC. Predictors used in the model are listed in Table S2 and included slope, a topographic roughness index (TRI), 30-year average climatic water balance deficit (DEF) and actual evapotranspiration (AET), 2001-2012 mean NDVI from MODIS, and gridded 1 km STATSGO available water holding capacity. The fitted random forest model explained 55% of the total variance in gSSURGO AWC. Partial response plots showing the conditional response of AWC to each predictor variable are shown in Figure A1.1. The raw and model infilled AWS grids are shown in Figure A.1.2.

Table A1.2. Predictor variables used to model missing gSSURGO data.

| **Variable** | **Description** | **Source** | **resolution** | **Units** |
| --- | --- | --- | --- | --- |
| AWC | STATSGO water holding capacity | STATSGO | 1 km | mm |
| ELEV | elevation | National Elevation Data | 240 m | meters |
| TRI | Terrain roughness index | National Elevation Data | 240 m | unitless |
| SLOPE | Slope (percent) | National Elevation Data | 240 m | % |
| DEF | Water balance deficit (1981-2010) | Dobrowski (2013) | 800 m | mm |
| AET | evapotranspiration | Dobrowski (2013) | 800 m | mm |
| NDVI | MODIS mean NDVI | NASA/MODIS | 240 m | unitless |
| SRAD | Clear sky July solar | This study | 240 m | W/m^2^ |


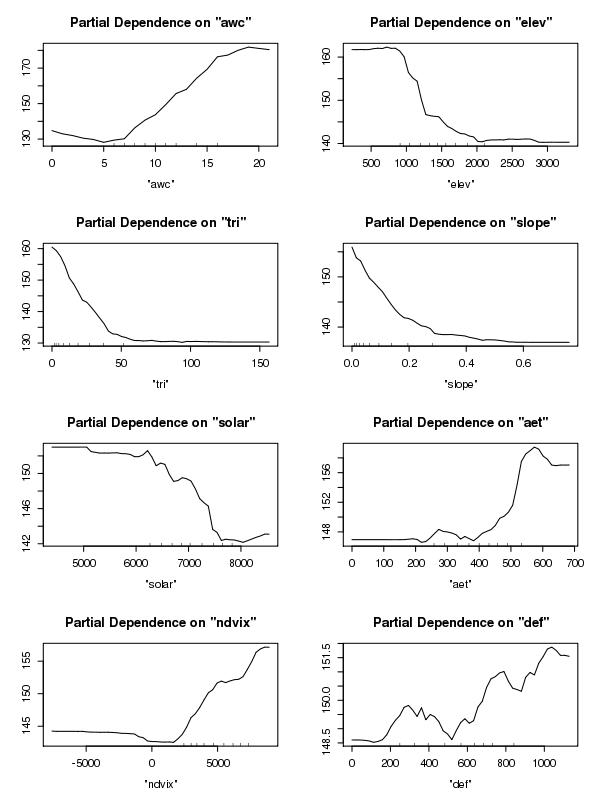


Figure A1.1. Partial response curves from the random forest regression model used to infill missing data in gSSURGO. These plots illustrate the relationships between topography and soil depth, with generally deeper soils on north slopes, shallow soils on steep slopes, and shallow soils on ridge tops. Shallow rocky soils at higher slope positions and ridgetops should be particularly important for WBP.


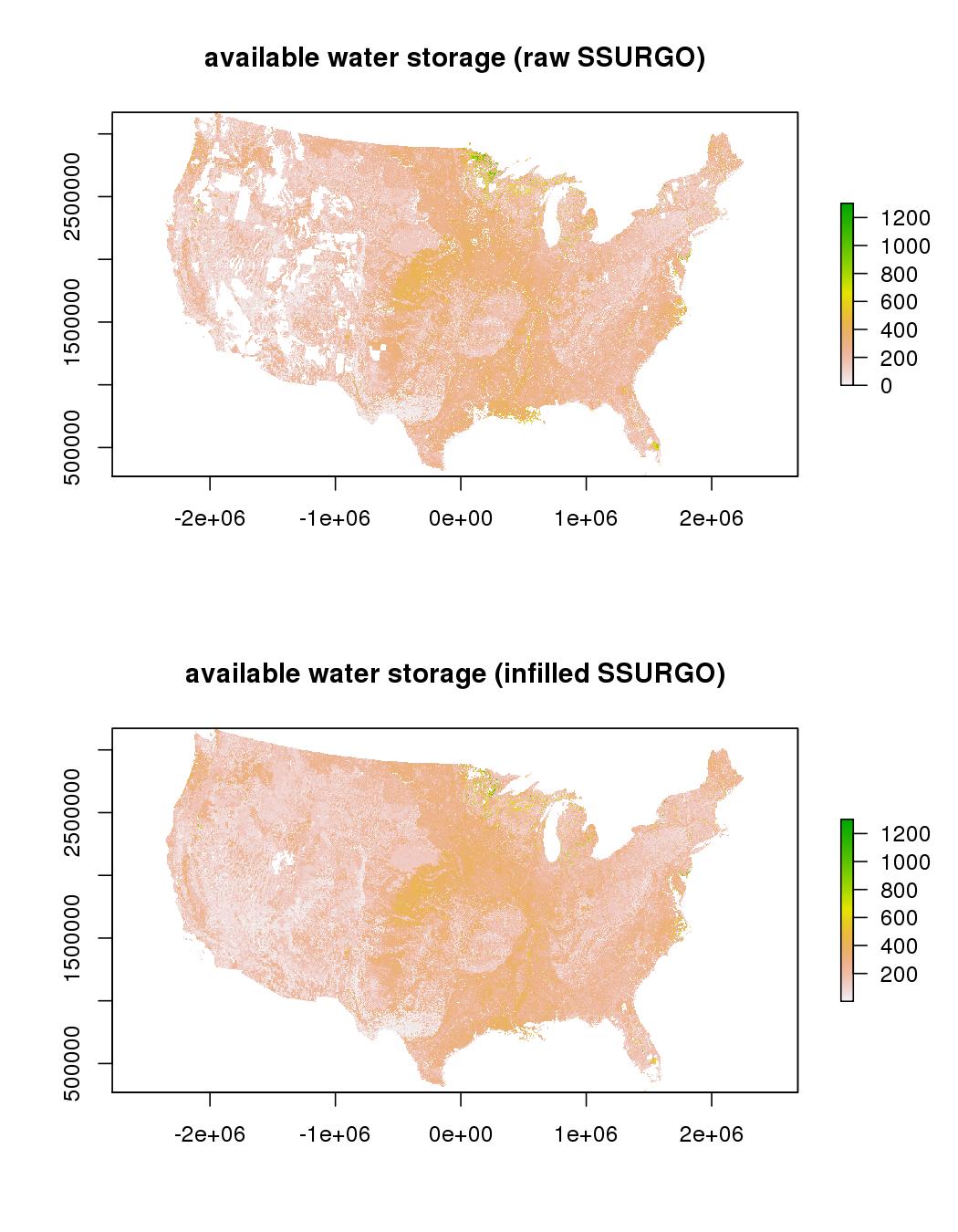


Figure A1.2. 250 m gridded SSURGO Available Water holding Capacity (AWC) prior to infilling (top panel) and model infilled (bottom panel).

**References**

# Holden Z.A., A. Swanson, A. Klene, J. Abatzoglou, S. Dobrowski, S. Cushman, J. Squires, G. Moisen and J. Oyler (2015). Development of high-resolution (250 m) historical daily gridded air temperature data using reanalysis and distributed sensor networks for the US Northern Rocky Mountains. Int. J. Climatol. doi: 10.1002/joc.4580

Dobrowski, S., J. Abatzoglou, A. Swanson, J. Greenberg, A. Mysneberge, Z.A. Holden and M. Schwartz (2012). The climate velocity of the contiguous United States during the 20th century. Global Change Biology.19:241-251.

Mesinger F, DiMego G, Kalnay E, Mitchell K, Shafran PC, Ebisuzaki W, Jovic D, Woollen J, Rogers E, Berbery E, Ek MB, Fan Y, Grumbine R, Higgins W, Li H, Lin Y, Mankin G, Parrish D, Shi W. 2006. North American regional reanalysis. Bull. Am. Meteorol. Soc. **87:** 343–360.

Forthofer, J. 2007. Modeling wind in complex terrain for fire spread prediction. Masters Thesis, Colorado State University. 123 pages.
