# Supplementary material for: Using Landscape Genetics Simulations for Planting Blister Rust Resistant Whitebark Pine in the US Northern Rocky Mountains
Source: Front Genet. 2017 Feb 10;8:9. doi: 10.3389/fgene.2017.00009 (PMC5300977; doi:10.3389/fgene.2017.00009)
Supplement: Supplementary file 2 [file DataSheet2.DOCX]

# **Appendix 2. Modeling regeneration potential for whitebark pine using a climatic water balance approach**

High resolution (240 m) 30-year normal (1981-2010) gridded temperature and water balance datasets described in Appendix 1 were used to predict the occurrence of juvenile (< 100 cm height) Whitebark Pine (WBP) across the US Northern Rockies. Six physically-based variables were used as predictors. These included 30-year average maximum and minimum temperature, 30-year average climate water balance deficit and evapotranspiration, 30-year average April Snow Water Equivalent (SWE) and mean annual solar insolation. Maps of predictor variables and the location of Forest Inventory and Analysis (FIA) plots used in the analysis are shown in Figure A2.1.

Conditional density plots showing the occurrence of WBP juveniles with respect to each predictor variable are shown in Figure A2.2. Dark gray areas denote regions in climate space with higher probability of occurrence. These plots generally suggests that this species prefers cold, low energy, relatively dry sites with relatively high insolation (south facing slopes). These relationships likely reflect the ability of WBP to tolerate harsh conditions (i.e., shallow soils near ridge tops) that limit competition from other species.

A stochastic gradient boosting model (GBM; Friedman 2001; Friedman 2002) was used to predict the presence or absence of juvenile WBP at all Region 1 FIA plots (N=4123). GBM is a form of machine learning algorithm that optimizes model fit based on iteratively training a model using withheld data. Model accuracy was assessed using a suite of accuracy statisticsthat include percent of plots correctly classified (PCC), area under the receiver operation curve (AUC) and Kappa statistic and are shown in Table A2.1. Overall, the model shows very good to excellent model fit, with 92% of plots correctly classified and area under the receiver operation curve (AUC) of 0.91. Partial response plots for the top 4 predictor variables and the top two strongest 2-way interactions are shown in Figure A2.3. Partial plots suggest a preference for sites with cold daytime and nighttime temperature and sites with high moisture deficit.

Table A2.1. Accuracy statistics for the model of juvenile whitebark pine occurrence.

| PCC | sensitivity | specificity | Kappa | AUC |
| --- | --- | --- | --- | --- |
| 0.92 | 0.25 | 0.97 | 0.30 | 0.91 |

|  |  |  |  |  |
| --- | --- | --- | --- | --- |


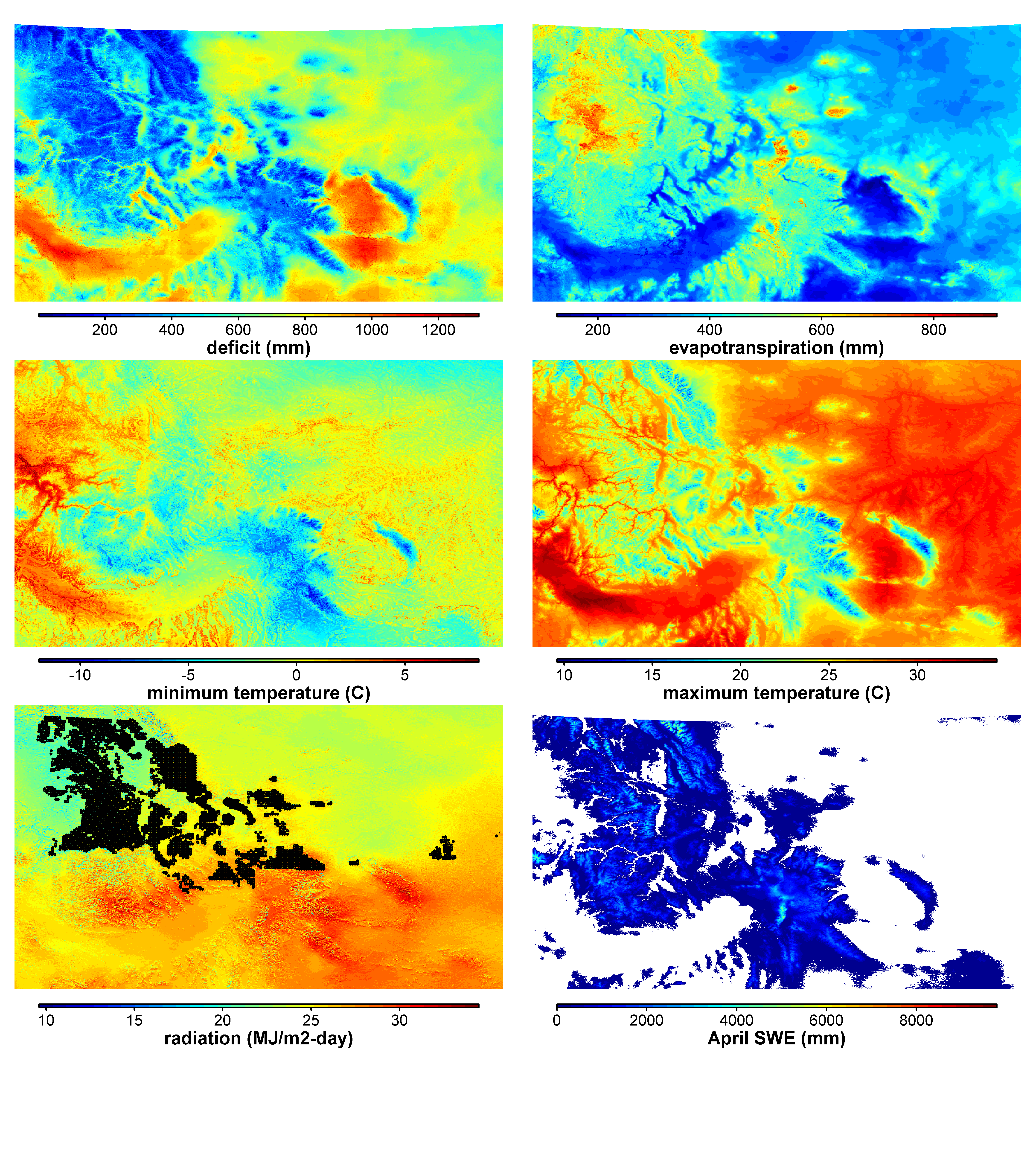


Figure A2.1. Temperature, water balance, radiation and snow predictors used to model the occurrence of juvenile (< 100 cm height) whitebark pine. FIA plot locations are shown in the bottom left panel.


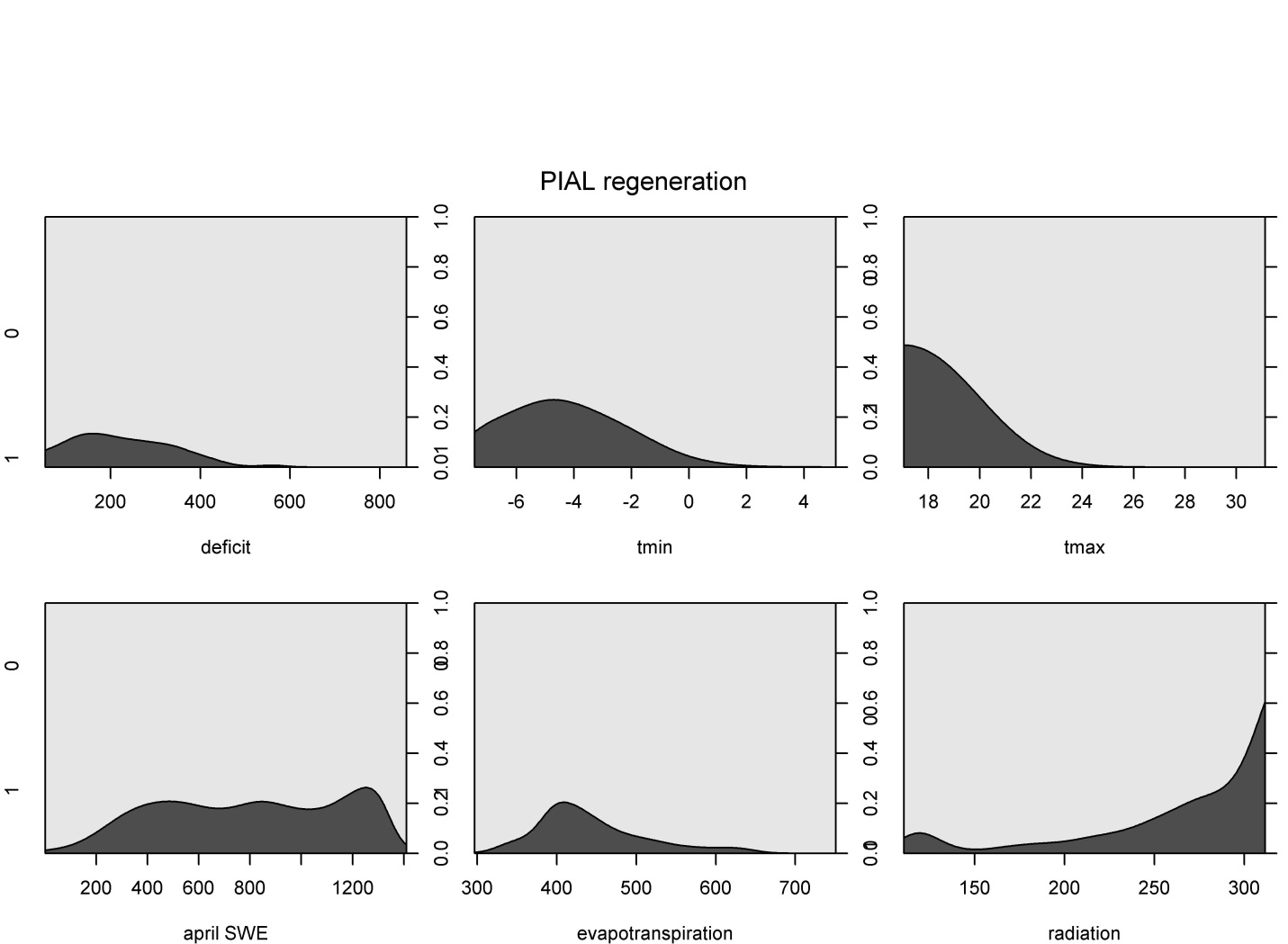


Figure A2.2. Conditional density plots for presence and absence of juvenile WBP at all FIA and intensified FIA grid plots (N=4123) on USDA Forest Service lands in the Northern Region. The plots indicate that WBP prefers cold, relatively dry, low energy sites with higher exposure to solar insolation.


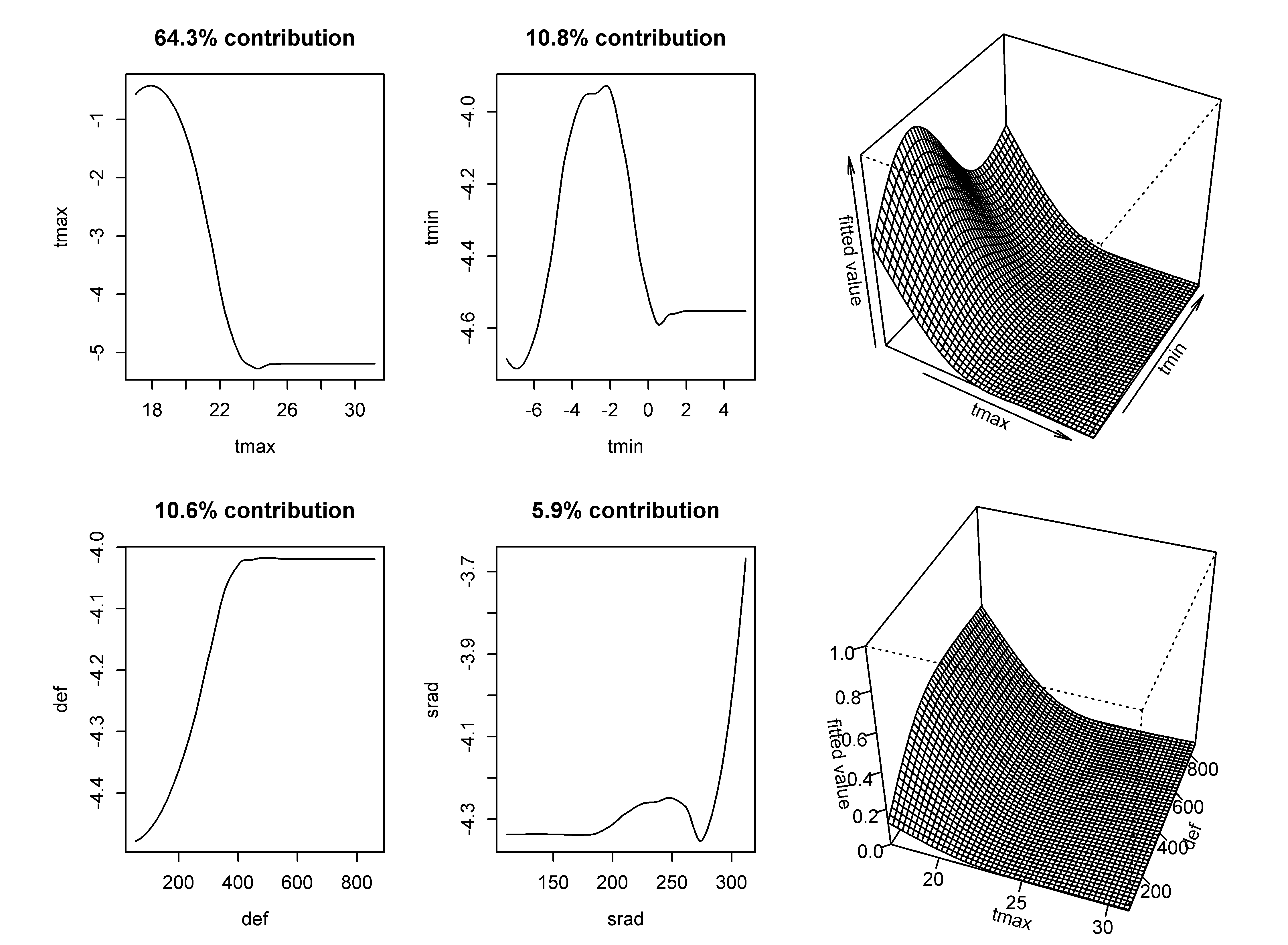


Figure A2.3. Partial response curves from a boosted regression tree model showing the response of whitebark pine juvenile presence conditional on each predictor variable.


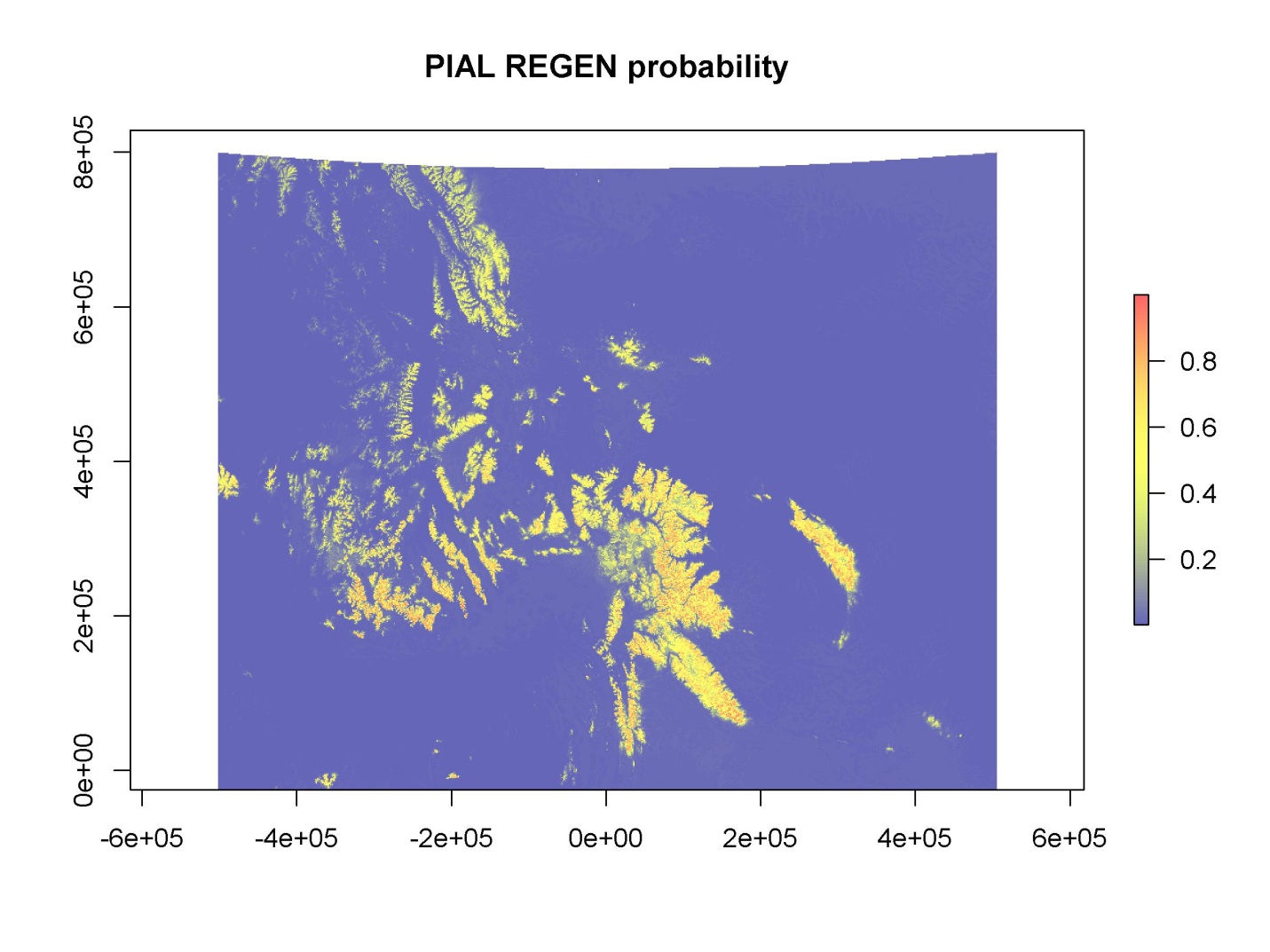


Figure A2.3. Prediction from a boosted regression tree model for juvenile whitebark pine showing the predicted probability of occurrence across the US Northern Rockies.

-
