# Supplementary material for: Using Landscape Genetics Simulations for Planting Blister Rust Resistant Whitebark Pine in the US Northern Rocky Mountains
Source: Front Genet. 2017 Feb 10;8:9. doi: 10.3389/fgene.2017.00009 (PMC5300977; doi:10.3389/fgene.2017.00009)
Supplement: Supplementary file 3 [file DataSheet3.DOCX]

# **Appendix 3. Development of a blister rust probability model for the US Northern Rocky Mountains**

Following methods described in Appendix A2 for modeling WBP occurrence, we developed a model of blister rust (BR) probability for the US Northern Rocky Mountains. Initially, two data sets were evaluated as sources of information about the occurrence of BR. First, Forest Inventory and Analysis (FIA) plots were queried for the occurrence of Blister Rust. All plots noting damage associated with rust fungus and containing either whitebark pine, limber pine or western white pine were counted as having BR present on the plot (n=380 plots ). Additionally, we evaluated the WILIS (aka “High-Five” database, which specifically recorded the presence and extent of mortality associated with BR infection. However, the majority of these plots lacked precisely georeferenced plot location information and were therefore unsuitable for extracting relatively fine-scale predictors. Therefore only FIA plots were used were not used in this analysis.

Reports in the literature note a relationship between air temperature, humidity and BR at various stages of its life cycle (Van Arsdel et al. 2006; Kearns 2005; Kearns and Jacobi 2006). Analysis of BR hazard in Colorado noted that disease incidence was higher given longer frost-free periods and warmer nighttime temperatures in September, and higher levels of precipitation in July. Generally, past studies suggest that BR prefers relatively cool, moist conditions. To better elucidate temperature controls on BR occurrence, 30-year normal (1981-2010) seasonal temperature maps were created and evaluated as predictors of BR occurrence. These included spring (March-May) and summer (June-August) minimum and maximum temperature. Conditional density plots showing the relationship between BR occurrence and eight climate/biophysical variables are shown in Figure. A3.1. Presence of BR shows a strong preference for regions with moderately cool spring and summer daytime temperature, and areas with low moisture deficit. Using the temperature, water balance, snow and radiation datasets described in Appendix A2. A Gradient boosting model was developed to predict the occurrence of BR following methods described in Appendix A2.

**Model results**

The relative importance of predictors from a gradient boosting model for BR are shown in Figure A3.2. Spring (March-May) daytime maximum temperature was the strongest predictor, followed by spring miminum temperature and summer (June-August) maximum temperature. These results support previous work suggesting that temperature and humidity may limit the range of BR occurrence.


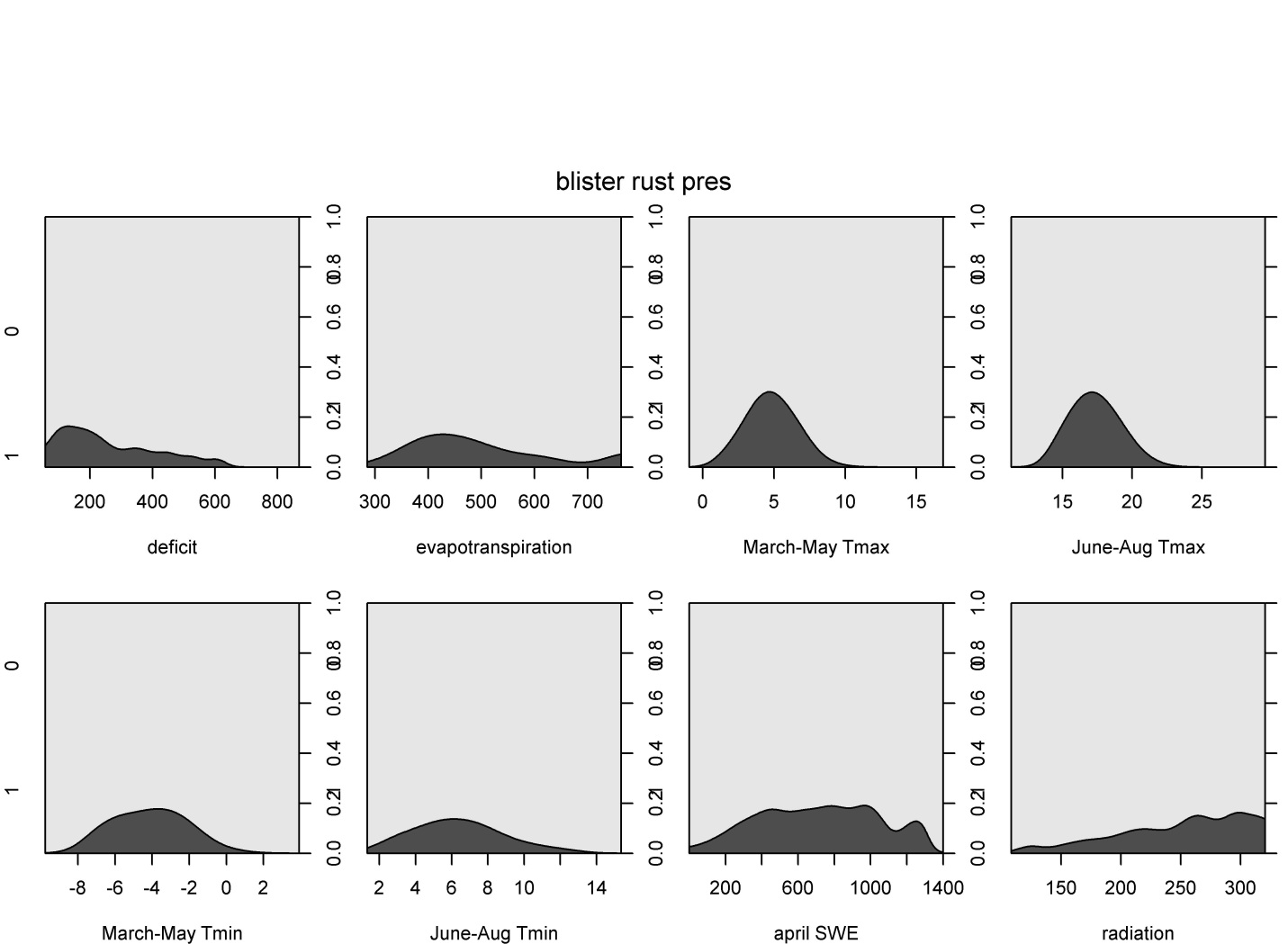


Figure A3.1. Conditional density plots for Blister Rust occurrence on 4,296 FIA plots. Dark gray areas denote regions in climate space with higher probability of occurrence for BR. Presence of BR shows a strong preference for regions with moderately cool spring and summer daytime temperature, and areas with low moisture deficit.


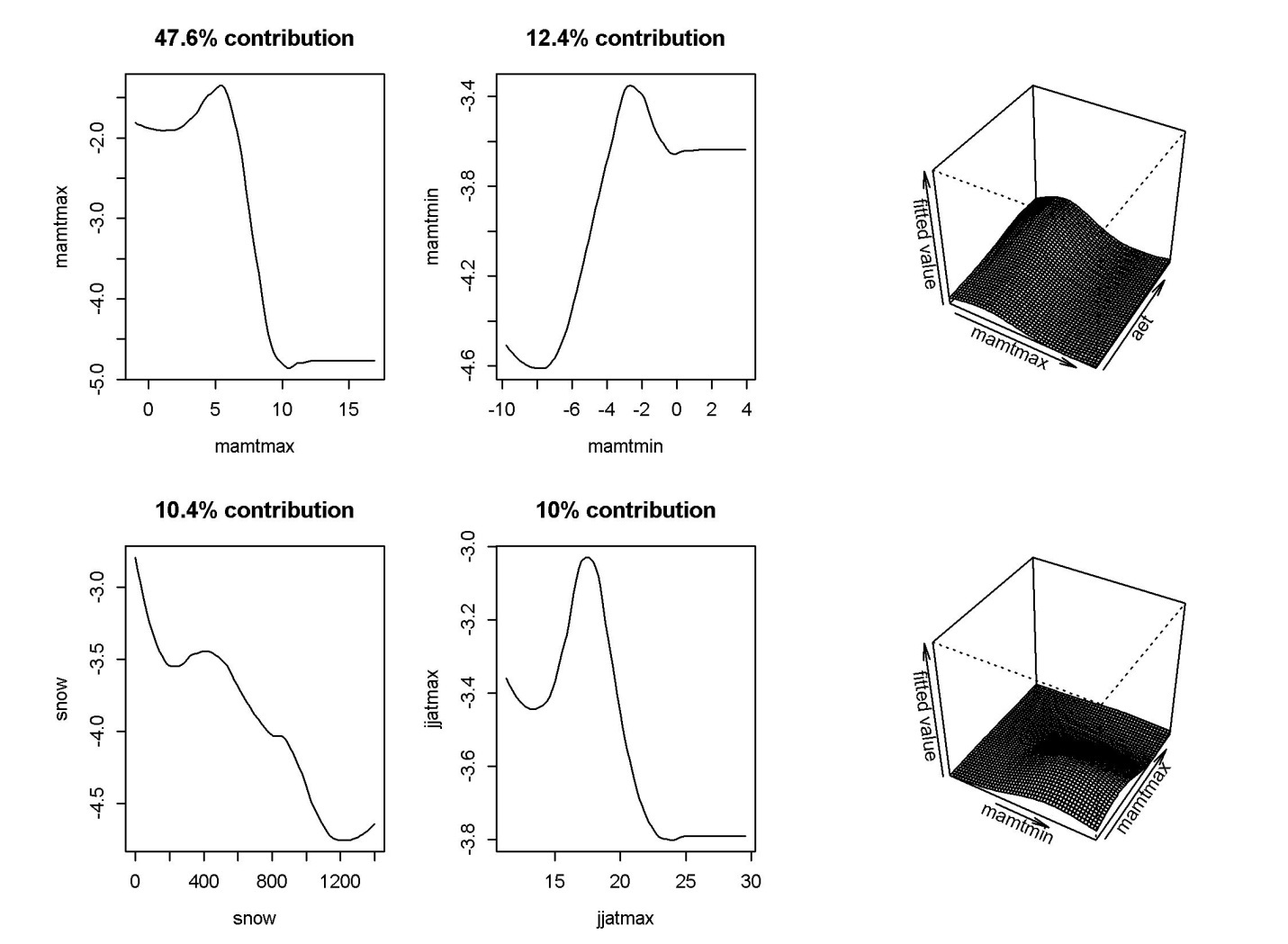


Figure A3.3. Partial response plots from a boosted regression tree model for BR with the top two 2-way interaction plots shown to the right.


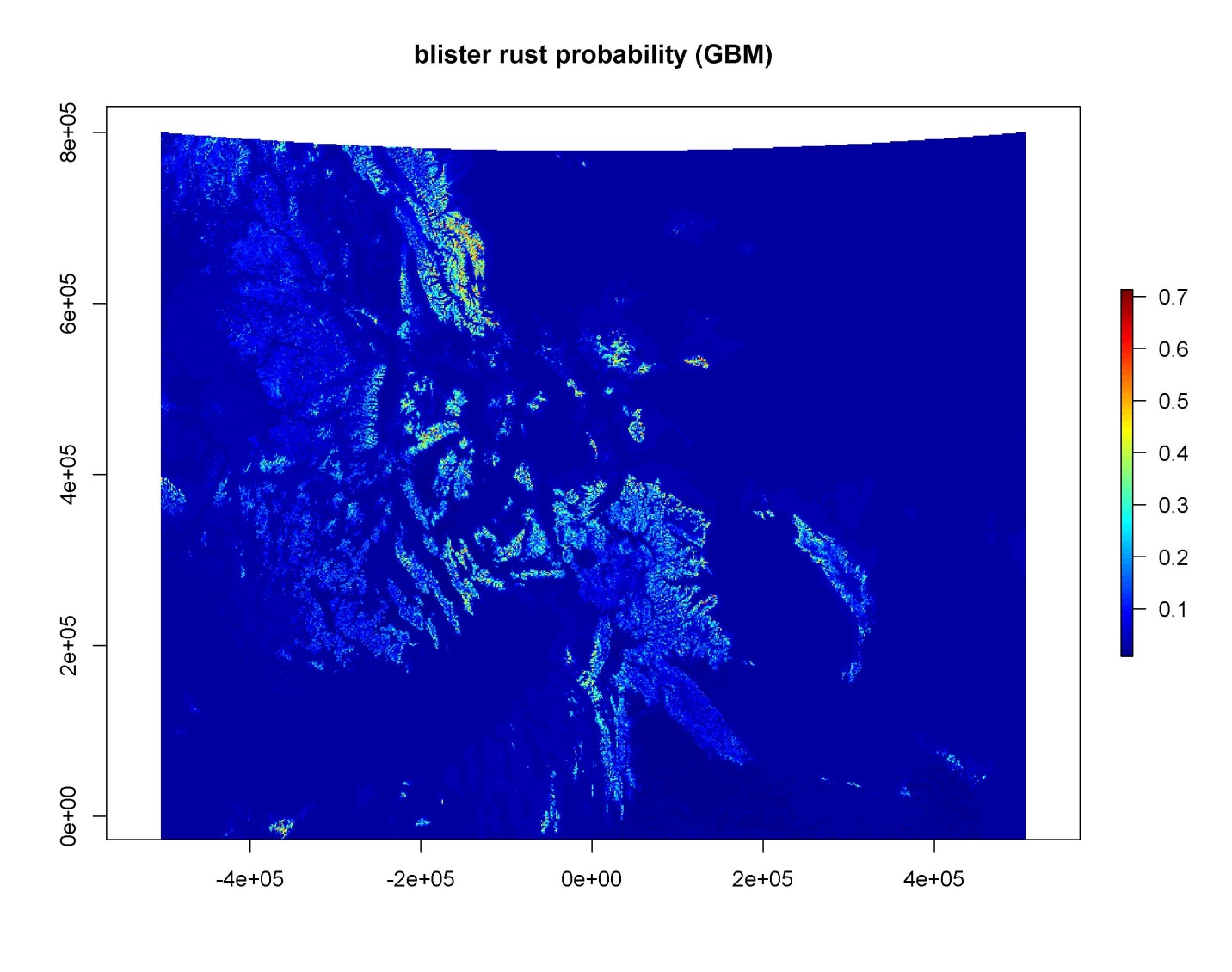


Figure A3.4. Predicted probability of occurrence for blister rust for the US Northern Rocky Mountains.

**References**

Kearns, H.S.J. 2005. White pine Blister Rust in the central Rocky Mountains: Modeling current status and potential impacts. Fort Collins, CO: Colorado State University. 243 p. Dissertation.

Kearns, H.S.J. and Jacobi, W. 2006. The distribution and incidence of white pine Blister Rust in central and southeastern Wyoming and northern Colorado. Can. J. Forest Res.

Van Arsdel, E., B.W. Geils, and P.J. Zambino (2006). Epidemiology for Hazard Rating of White Pine Blister Rust. In: In: Guyon, J. comp. 2006. Proceedings of the 53rd Western International Forest Disease Work Conference; 2005 August 26–29; Jackson, WY. Ogden, UT: U.S. Department of Agriculture, Forest Service, Intermountain Region.
