# Supplementary material for: Using Landscape Genetics Simulations for Planting Blister Rust Resistant Whitebark Pine in the US Northern Rocky Mountains
Source: Front Genet. 2017 Feb 10;8:9. doi: 10.3389/fgene.2017.00009 (PMC5300977; doi:10.3389/fgene.2017.00009)
Supplement: Supplementary file 4 [file DataSheet4.DOCX]

# **Appendix 4**. CDMetaPOP whitebark pine work flow and parameters

#
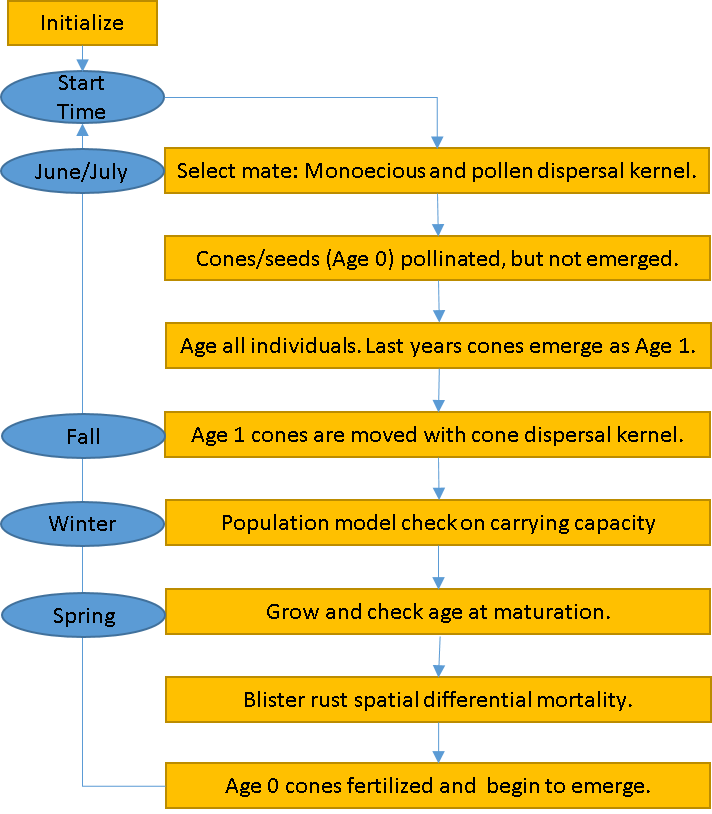


Figure A4.1. CDMetaPOP flow diagram with respect to Whitebark Pine major processes.

Table A4.1: Parameter list

| **Input parameter** | **Description** | **Values** | **References** |
| --- | --- | --- | --- |
| ***Patch-level controls*** | | | |
| **Patches, (X,Y)** | Number of patches and location | 1059 placed in 0.5 or higher probability of WBP occurrence | Section 2.1 |
| **K** | Carrying capacity | 100 individuals | - |
| **N** | Initial abundance | 50 individuals | - |
| **Genetics** | Initial allele frequency | 4 Zones (INLA, CLMT, BTIP, GYGT) | Mahalovich & Hopkins (2011) |
| **Mortality** | Patch-level mortality | 0 | - |
| **Migration probability** | Emigration probability | 1 | - |
| **Local dispersal probability** | Straying probability | 0 | - |
| **Temperature Winter** | Temperature values used to grow individuals | NA | - |
| **Grow Days Winter** | Grow days during this period | NA | - |
| **Temperature Summer** | Temperature values used to grow individuals | NA | - |
| **Grow Days Summer** | Grow days during this period | NA | - |
| **Locus AA spatial selection** | Patch-level mortality values for given genotype AA linked to Blister Rust resistance. | Genotype assumed to be under selection; see simulation scenarios | Section 2.1 |
| **Locus Aa spatial selection** | Patch-level mortality values for given genotype Aa linked to Blister Rust resistance. | Blister Rust mortality values used; see simulation scenarios. | - |
| **Locus aa spatial selection** | Patch-level mortality values for given genotype aa linked to Blister Rust resistance. | Blister Rust mortality values used; see simulation scenarios. | - |
| ***Class-level controls*** | | | |
| **Age class** | Number of age classes | 500 (Age 0 = seeds; Age 1 = cones) | - |
| **Body size** | Size (DBH) of each age class | 0.2 cm incremental growth each year | Keane et al. 2007 |
| **Distribution** | Initialize size classes within stands | Initialize random (each age has equal probability) | - |
| **Mortality** | Class-specific mortality | Cumulative sapling mortality until 35% survival for ages 1 - 15 | Izlar 2002 |
| **Migration probability** | Emigration probability | Age 1 'cones' = 1, else 0 | Owens et al. 2008 |
| **Local dispersal probability** | Straying probability | NA | - |
| **Maturation** | The probability of being a reproducing individual. | Age 20 and older are considered mature | Fire Effects Information System, accessed September 2015 |
| **Fecundity** | Seeds produced for each size class | BA m^2 = 0.00007854 * DBH^2; 2BA / ha yields 1000cones /ha = 500cones/1BA; 20 seed per cone | Keane et al. 2007 |
| **Size Control** | Processes can either operate based on size or age relationships | Age control | - |
| ***Run parameters and output*** | | | |
| **MCruns** | Replicate runs | 10 | - |
| **Runtime** | Total years | 130 | - |
| **Start genes** | Year at which genetic exchange begins | 25 | - |
| **Start selection** | Year at which spatial selection begins | 25 | - |
| **Output years** | Years individual data produced | 0,24,25,30,35,40,50,60,70,80,90,100,110,120,130 | - |
| **Output format** | Format genetic output | GENALEX | - |
| **Population model** | Population growth model choice | Density independent model | - |
| ***Landscapes and movement rules*** | | | |
| **Pollen surface and movement rules** | Resistance to movement surface and kernels | Isolation-by-distance; inverse square probability 1/ Euclidean distance ^2 with maximum movement at 450 km.  Isolation-by-wind resistance; iverse square probability 1/Wind distance ^2 with maximum movement at 450 km. | UNICOR (Landguth et al. 2010) |
| **Seed surface and movement rules** |  | Isolation-by-distance; inverse square probability 1/ Euclidean distance ^2 with maximum movement at 30 km | Lorenz et al. 2011 |
| ***Reproduction options*** | | | |
| **Sexual reproduction** | Heterosexual or mononecious reproduction | monoecious | - |
| **Selfing** | Consider selfing | Yes | - |
| ***Offspring options*** | | | |
| **Offno** | Draw choice for seeds produced | Poisson | - |
| **Equal Clutch Size** |  | Yes | - |
| **Masting** |  | No | - |
| **Seed mortality** |  | 99% | DeMastus 2013 |
| ***Genetic options*** | | | |
| **Loci/alleles** | Number of loci/alleles | 17/9 | Mahalovich & Hipkins (2011) |
| **Mutation rate and model** |  | 0 | - |
| ***Spatial selection options*** | | | |
| **Type of selection** | Selection model | Selection applied to mature individuals only | - |
| **Implement selection** | The time of year to apply spatial selection | See time flow diagram | - |
| ***Growth options*** | | | |
| **Growth option** | Model for incremental growth for each individuals | Known increment of DBH = 0.2 cm per year. | Keene et al. 2007 |


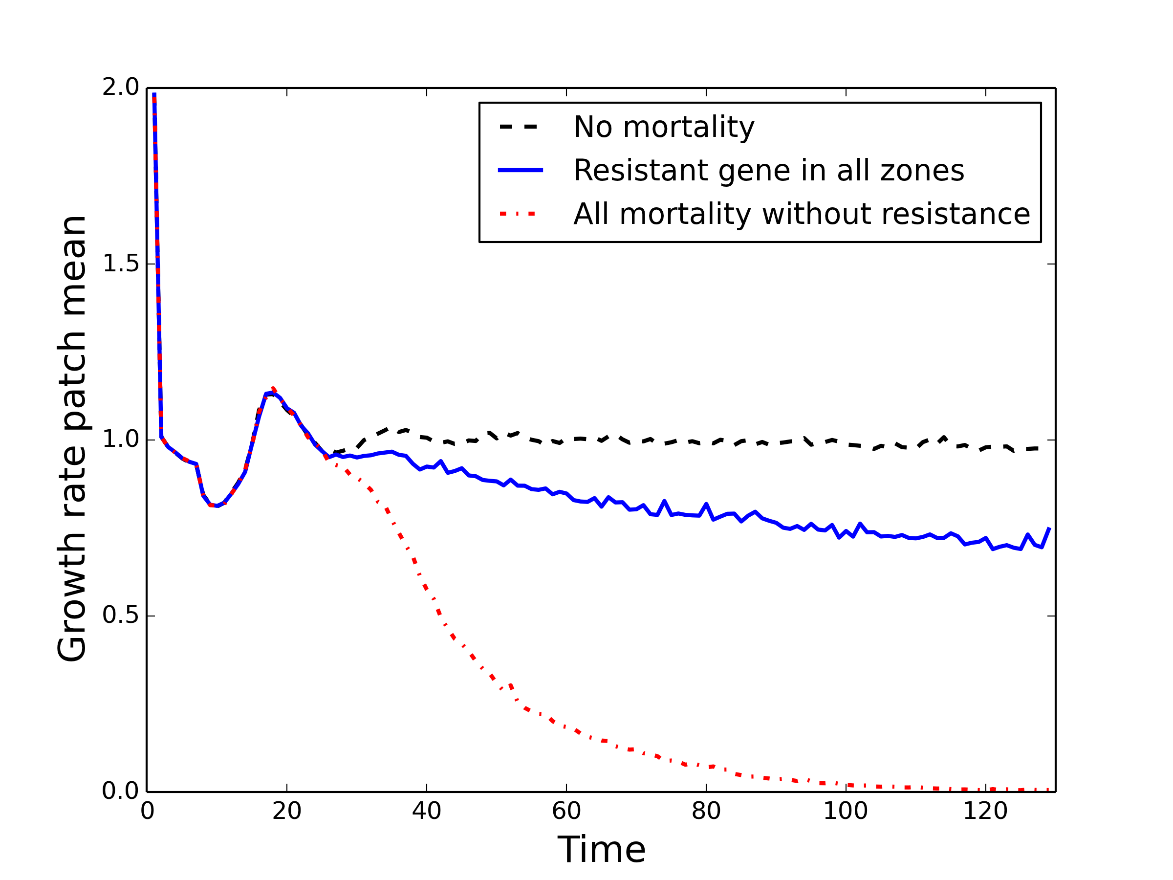


Figure A4.2a. Patch (stand) mean growth rate for each scenario in Block 1.


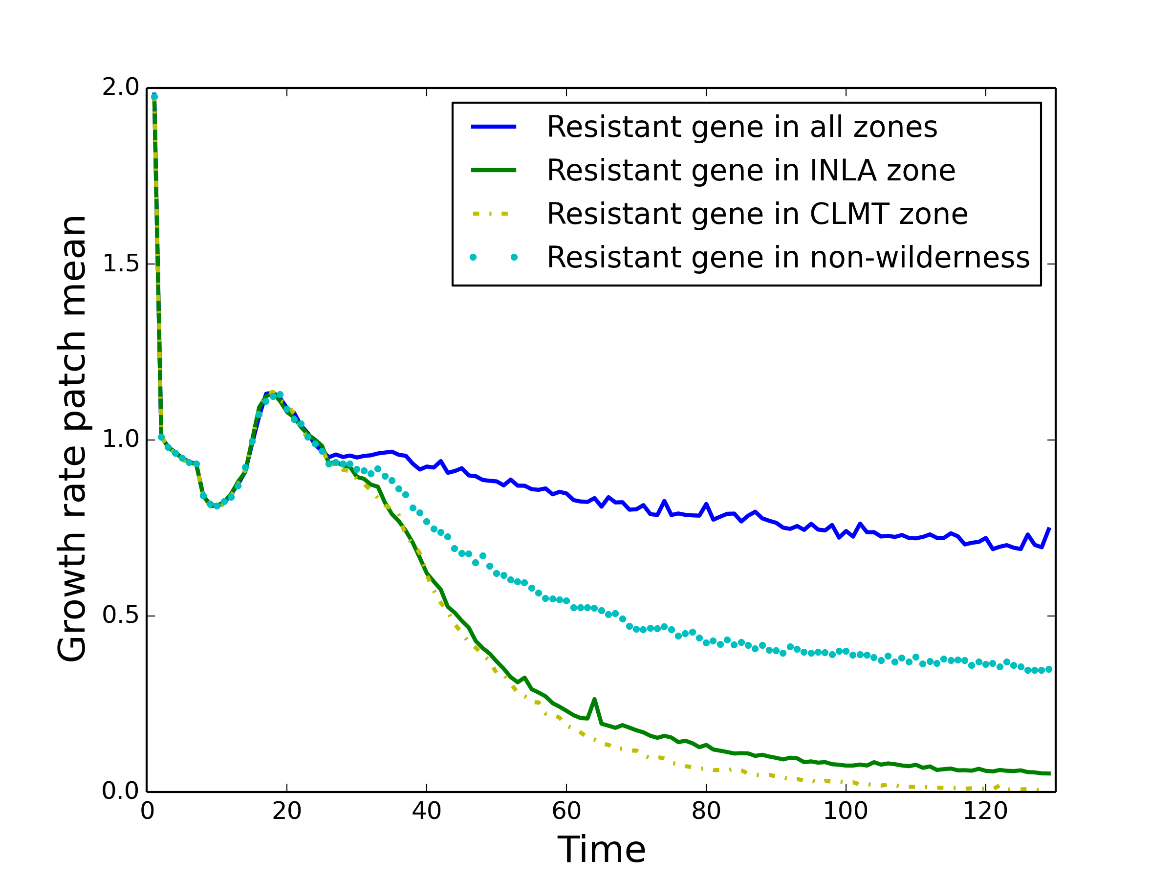


Figure A4.2b. Patch (stand) mean growth rate for each scenario in Block 2.


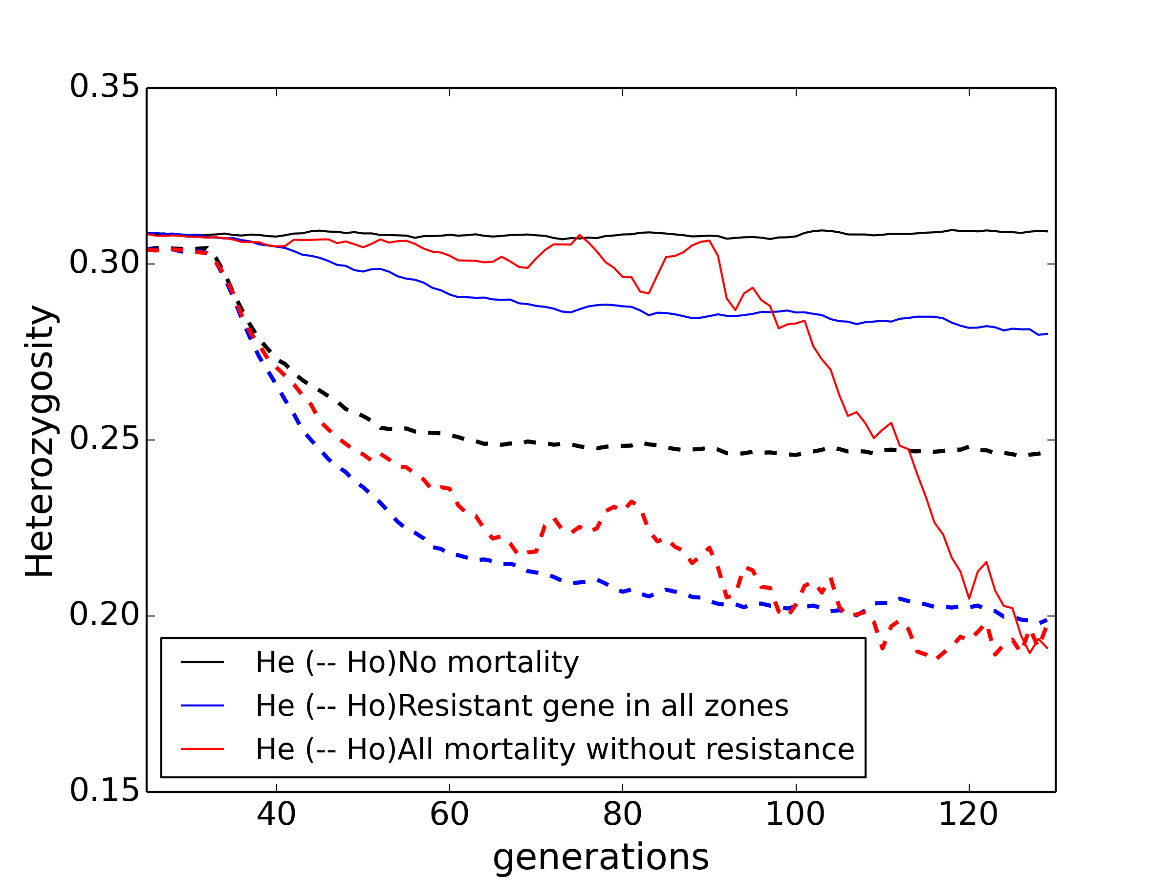


Figure A4.3a. Population measures of heterozygosity for each scenario in Block 1. Solid lines indicate expected heterozygosity and dash-dotted lines show observed heterozygosity.


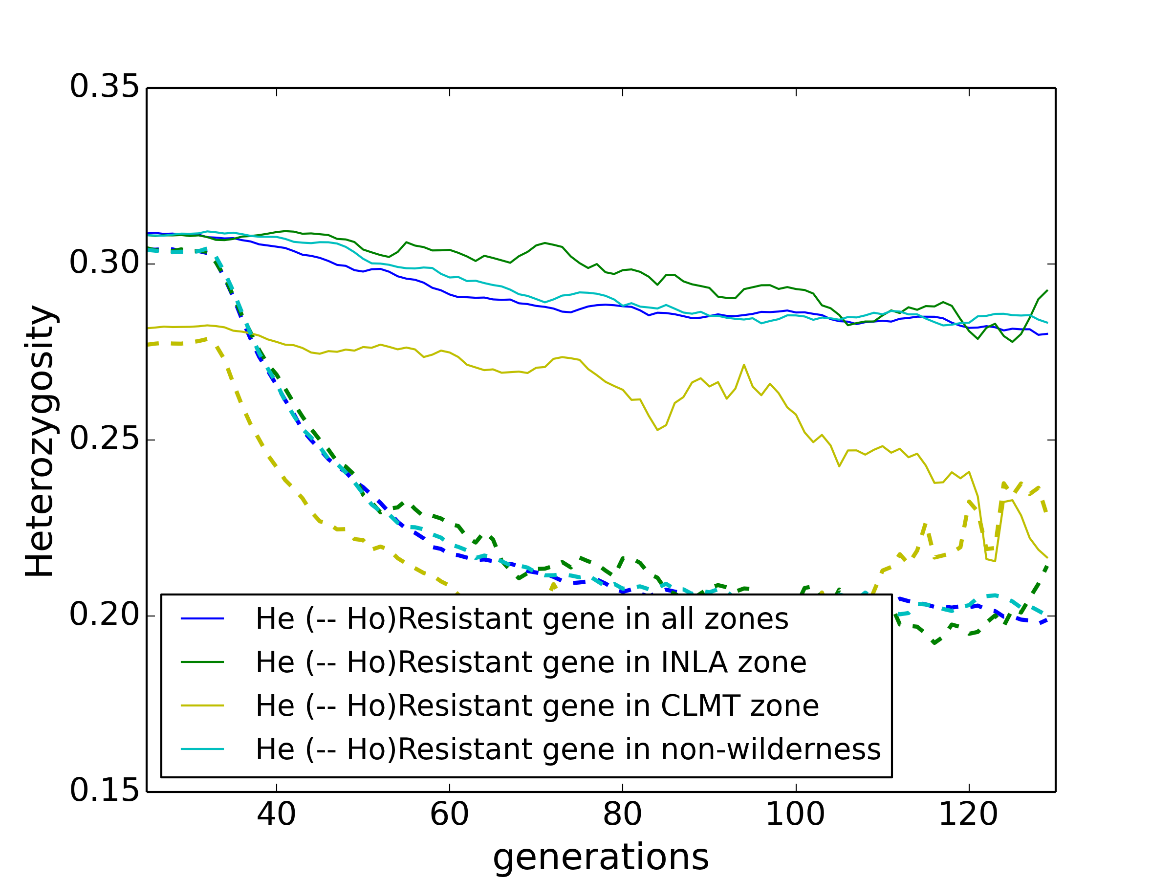


Figure A4.3b. Population measures of heterozygosity for each scenario in Block 2. Solid lines indicate expected heterozygosity and dash-dotted lines show observed heterozygosity.


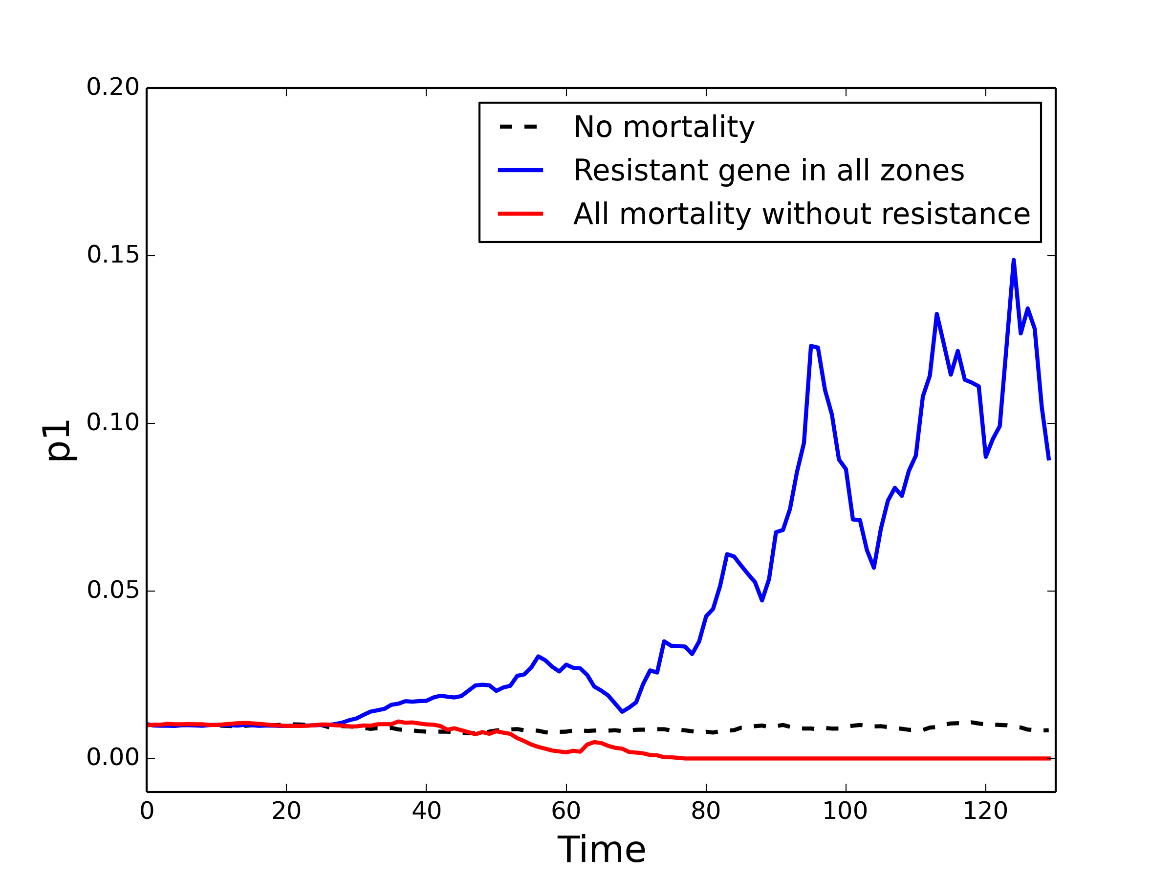


Figure A4.4a. Frequency of allele under selection in ‘Resistant gene in all zones’ scenario (blue solid line) and in scenarios in which the allele is not under selection (‘No Mortality’ and ‘All Mortality’) scenarios (black dashed line and red solid line, respectively).


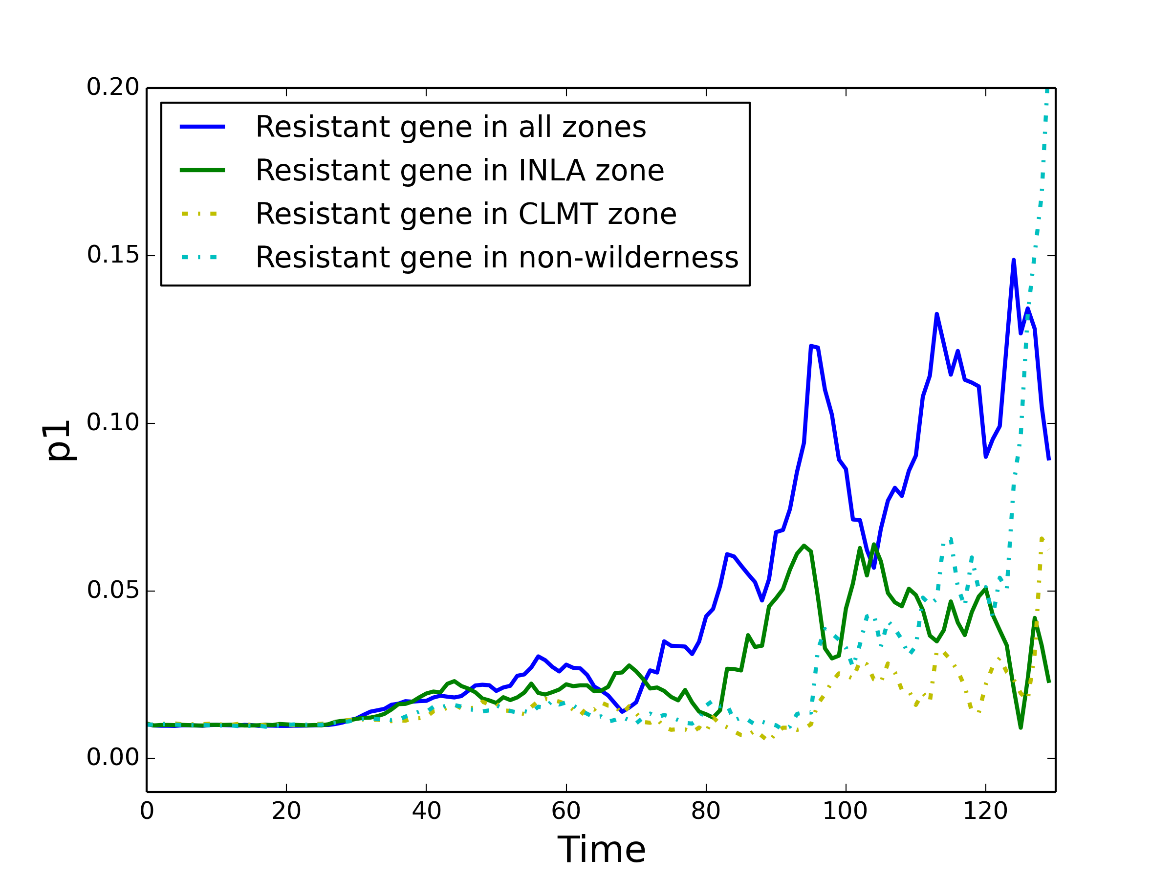


Figure A4.4b. Frequency of allele under selection in all scenarios for Block 2.

**References**

DeMastus CR (2013) Effective methods of regenerating whitebark pine (Pinus albicaulis)

through direct seeding (Doctoral dissertation, Montana State University-Bozeman, College of Letters & Science).

Izlar DK (2002) Assessment of whitebark pine seedling survival for Rocky Mountain

plantings. M.S. Thesis Humboldt State Univeristy.

Keane R, Gray K, Dickinson L (2007). Whitebark pine Diameter Growth Response to Removal

of Competition. RMRS General Technical Report Research note RN-32.

Landguth EL, Hand BK, Glassy JM, Cushman SA, Sawaya M (2012) UNICOR: a

species corridor and connectivity network simulator. Ecography, 12, 9-14.

Lorenz TJ, Sullivan KA, Bakian AV, Aubry CA (2011) Cache-site selection in Clark’s

nutcracker (*Nucifraga columbiana*). Auk. 128(2): 237−247.

Mahalovich MF, Hipkins VD (2011) Molecular genetic variation in whitebark pine (Pinus

albicaulis Engelm.) in the Inland West. In: Keane, R.E. (ed.) "High-Five Symposium: The Future of High-Elevation Five-Needle white pines in Western North America. 2010 June 28-30, Missoula, MT, USA. Proc: RMRS-P-63. For Collins, CO: USDA Forest Service, Rocky Mountain Research Station, p. 124-139.

Owens, J. N., Kittirat, T., & Mahalovich, M. F. (2008). whitebark pine (*Pinus albicaulis* Engelm.) seed production in natural stands. *Forest Ecology and Management*, *255*(3), 803-809.
